# ACSS2-dependent histone acetylation improves cognition in mouse model of Alzheimer’s disease

**DOI:** 10.1186/s13024-023-00625-4

**Published:** 2023-07-12

**Authors:** Yingbin Lin, Anlan Lin, Lili Cai, Weibin Huang, Shanzhi Yan, Yuanxiang Wei, Xinglin Ruan, Wenting Fang, Xiaoman Dai, Jinbo Cheng, Jie Zhang, Wanjin Chen, Qinyong Ye, Xiaochun Chen, Jing Zhang

**Affiliations:** 1 Department of Neurology, Fujian Medical University Union Hospital, Fujian Key Laboratory of Molecular Neurology and Institute of Neuroscience, Fujian Medical University, Fuzhou, China; 2 Present Address: Department of Neurology and Neurosurgery, Institute of Neurology, The First Affiliated Hospital of Fujian Medical University, Fuzhou, China; 3 The School of Basic Medical Sciences, Fujian Medical University, Fuzhou, China; 4 Beijing Institute of Basic Medical Sciences, Beijing, China

**Keywords:** Alzheimer’s disease, ACSS2, Synaptic plasticity, Histone acetylation, Acetate, Glutamate receptors

## Abstract

**Background:**

Nuclear acetyl-CoA pools govern histone acetylation that controls synaptic plasticity and contributes to cognitive deterioration in patients with Alzheimer’s disease (AD). Nuclear acetyl-CoA pools are generated partially from local acetate that is metabolized by acetyl-CoA synthetase 2 (ACSS2). However, the underlying mechanism of histone acetylation dysregulation in AD remains poorly understood.

**Methods:**

We detected ACSS2 expression and histone acetylation levels in the brains of AD patients and 5 × FAD mice. When we altered ACSS2 expression by injecting adeno-associated virus into the dorsal hippocampus of 5 × FAD mice and replenished ACSS2 substrate (acetate), we observed changes in cognitive function by Morris water maze. We next performed RNA-seq, ChIP-qPCR, and electrophysiology to study molecular mechanism underlying ACSS2-mediated spatial learning and memory in 5 × FAD mice.

**Results:**

We reported that ACSS2 expression and histone acetylation (H3K9, H4K12) were reduced in the hippocampus and prefrontal cortex of 5 × FAD mice. Reduced ACSS2 levels were also observed in the temporal cortex of AD patients. 5 × FAD mice exhibited a low enrichment of acetylated histones on the promoters of NMDARs and AMPARs, together with impaired basal and activity-dependent synaptic plasticity, all of which were rescued by ACSS2 upregulation. Moreover, acetate replenishment enhanced ac-H3K9 and ac-H4K12 in 5 × FAD mice, leading to an increase of NMDARs and AMPARs and a restoration of synaptic plasticity and cognitive function in an ACSS2-dependent manner.

**Conclusion:**

ACSS2 is a key molecular switch of cognitive impairment and that targeting ACSS2 or acetate administration may serve as a novel therapeutic strategy for the treatment of intermediate or advanced AD.

**Graphical Abstract:**

Nuclear acetyl-CoA pools are generated partly from local acetate that is metabolized by acetyl-CoA synthetase 2 (ACSS2). Model depicts that ACSS2 expression is downregulated in the brains of 5×FAD model mice and AD patients. Of note, ACSS2 downregulation mediates a reduction in ionotropic glutamate receptor expression through histone acetylation, which exacerbates synaptic plasticity impairment in AD. These deficits can be rescued by ACSS2 upregulation or acetate supplementation (GTA, an FDA-approved food additive), which may serve as a promising therapeutic strategy for AD treatment.

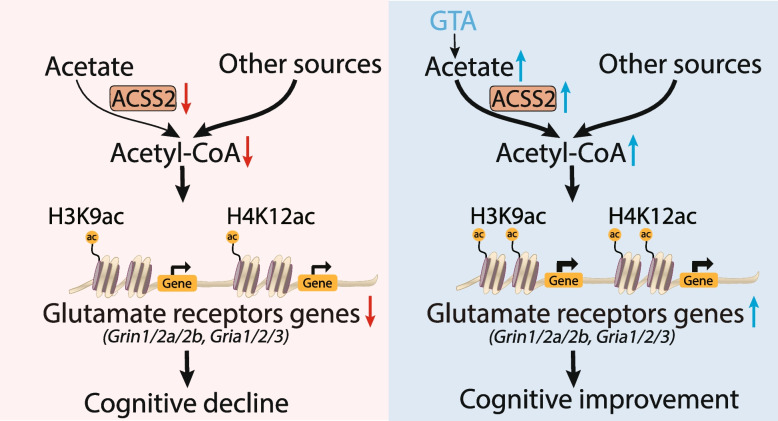

**Supplementary Information:**

The online version contains supplementary material available at 10.1186/s13024-023-00625-4.

## Background

As the most common type of dementia, Alzheimer’s disease (AD) is undoubtedly one of the most burdensome diseases of the twenty-first century [1]. In its pathogenesis, the disease has taken an insidious course, with amyloid-β (Aβ), essential for AD diagnosis, accumulating at least 20 years prior to the onset of any observable symptoms [2]. Therefore, in addition to early diagnosis and early intervention, it is of great urgency and significance to explore effective treatments for patients with mid-to-late AD [3]. During the long interval between Aβ accumulation and appearance of noticeable symptoms, in addition to tau pathology [4] and glia reaction [5], the impairment of synaptic plasticity [6–8] and loss of synapses [7–10] are the crucial factors for clinical AD progression from the pre-symptomatic stage to mild cognitive impairment (MCI) to dementia. Therefore, an exploration of the potential underlying mechanisms that can be exploited to restore the impaired synaptic plasticity in AD brain holds great promises for AD treatment.

Epigenetic regulation and chromatin remodeling have been reported to participate in the regulation of numerous neuronal functions, ranging from synaptic plasticity to learning and memory [11–16]. As a kind of chromatin remodeling, histone acetylation weakens the electrostatic affinity between histones and DNA, thus promoting the fundamental gene transcription for long-term synaptic plasticity and memory [17–21]. In the hippocampus of normal aging mice, histones H2B/H4 failed to be acetylated during learning, rendering them incapable of initiating the expression of memory consolidation-related genes [22, 23]. In the brain of APP/PS1 transgenic mice (a mouse model of AD), the acetylation of histone H3 [24] and H4 [25] is significantly downregulated. Similar findings have been reported in the postmortem brain tissues from AD patients [26, 27]. Several recent studies have proposed modifying histone acetylation as a promising therapeutic strategy for AD [25, 28]. However, due to specificity, efficacy, and safety issues, the insights gleaned from classic AD animal models have rarely been translated into practical clinical trials, despite the capability of histone-deacetylase (HDACs) inhibitors to ameliorate cognitive impairment [29, 30]. Thus, an alternative exploration into histone acetylation, such as a probe into the source of acetyl donor, may provide a better candidate for the restoration of synaptic plasticity in AD treatment.

As the acetyl donor, acetyl coenzyme A (acetyl-CoA), generated by the metabolic enzymes in the nucleus, may directly affect histone acetylation [31, 32]. So far, three principal enzymes have been shown to be crucial for maintaining nuclear acetyl-CoA levels in supporting histone acetylation: acyl-CoA synthetase short-chain family member 2 (ACSS2), ATP-citrate lyase (ACLY), and pyruvate dehydrogenase complex (PDC) [20, 32, 33]. Of interest, ACSS2, which catalyzes the synthesis of acetyl-CoA from acetate, can be recruited to the loci of memory-related neuronal genes to maintain a local acetyl-CoA pool, providing the substrate for histone acetylation and promoting the expression of specific genes, which is essential for maintaining long-term spatial memory [20]. In addition, ACSS2 is required for alcohol-induced neuron-specific transcription and alcohol-related associative learning [34]. Taken together, these findings underscore the pivotal role of ACSS2 in regulating neuronal histone acetylation and mammalian spatial memory. However, the role of ACSS2 in the aberrant histone acetylation of AD brain remains unknown.

In this study, we focused on key molecules involved in the impaired synaptic plasticity in the middle and late stages of AD. Our objective was to determine whether ACSS2 mediates the loss of synaptic plasticity in the AD brain and whether supplementation of ACSS2 substrate (acetate) can enhance synaptic plasticity and improve cognition. We also investigated the mechanisms for the actions of ACSS2 and acetate, which are largely unexplored in the context of AD.

## Methods

### Study design

The current study aimed to investigate the potential of ACSS2 upregulation and GTA administration (an FDA-approved food additive) in restoring synaptic plasticity and the underlying molecular mechanisms in the late-stage AD. We utilized an AD mouse model of both sexes at 8-10 months of age, an age with apparent cognition impairment. The research objectives, subjects of investigation, and overall experimental design were all described in Results section. Briefly, behavioral, electrophysiological, bulk RNA-sequencing, CHIP-PCR, and biochemical methods were employed to verify whether the upregulation of ACSS2 in the dorsal hippocampus and the systemic administration of GTA would consistently improve the synaptic plasticity of 5 × FAD mice. For all behavioral testing, electrophysiological recordings, and biochemical assays, experimenters were blinded to the genotype and treatment of mice. For behavioral testing and electrophysiological recordings, the testing order of mice was randomized. For statistical analyses, no data were excluded from behavioral testing, except when mice were found with obvious health problems, such as skin lesions, eye injuries, tumors, or slowed movements. In all experiments, data obtained from individual mice were considered one biological replicate. Replication and sample sizes for all experiments are detailed in the figure legends.

### Animals and human post-mortem tissues

The 5 × FAD mice, a model that co-expresses five familial Alzheimer’s disease mutations on human amyloid precursor protein [K670N/M671L (Swedish) + I716V (Florida) + V717I (London)] and human presenilin 1 (M146L + L286V) under the control of the murine Thy-1 promoter, were purchased from Jackson Laboratory (stock no. 034848-JAX, Bar Harbor, ME, USA). Genotypes were identified by PCR analysis of the tail DNA as previously described [35]. Both male and female mice (littermates of 2, 5, 8 and 10 months of age) were utilized. Similar numbers of male and female mice were used in each experimental group. The animals were housed in groups of no more than six mice per cage and maintained on a standard 12-h light/dark cycle at 22 ± 1 °C. All animal studies were performed in compliance with the rules and regulations of the Institutional Animal Care and Use Committee at Fujian Medical University and followed the international guidelines for the ethical use of animals.

Frozen postmortem brain tissues from patients with AD and normal control subjects were provided by National Human Brain Bank for Development and Function, Chinese Academy of Medical Sciences, and Peking Union Medical College, Beijing, China. The detailed demographic and clinical information has been described in Appendix Table S2. The experimental procedures involving frozen postmortem brain tissues were approved by the Ethics Board at Institute of Basic Medical Sciences, Chinese Academy of Medical Sciences and Peking Union Medical College (approval no. 009-2014). After quick-freezing with liquid nitrogen, the brain tissues were stored at -80 °C until further processing. Frozen human brain samples were used for ACSS2 protein detection.

### Drug

Glyceryl triacetate (GTA) was purchased from Sigma-Aldrich (#90240) and dissolved in normal saline. The dose of GTA (2 g /kg /day) was adjusted according to our previous studies [36].

### Stereotaxic virus injection

The 5 × FAD and wildtype (WT) mice (aged 7-8 months old) were anaesthetized with isoflurane and placed on a stereotactic frame (stoelting, USA). A small volume of virus was injected bilaterally into the CA1 area of the dorsal hippocampus (AP, − 1.9 mm from bregma; DV,**-**1.15 mm from skull surface; ML, ± 1.45 mm from midline) using a pulled glass capillary with a pressure microinjector at a slow rate of 50 nl/min. After viral injection, the capillary was left for an additional 5 min and then slowly removed.

The following vectors were used: *Acss2* overexpression vector (rAAV-CMV-*Acss2*-HA-P2A-EGFP-WPRE-pA) and eGFP control vector (rAAV-CMV-HA-P2A-EGFP-WPRE-pA) were designed, validated, and synthesized by Shumi Brain Science and Technology Co., LTD (Wuhan, China); *Acss2* knockdown vector (pHBAAV2/9-U6-m-*Acss2* shRNA-CMV-EGFP) and eGFP control vector (pHBAAV2/9-U6-MCS-CMV-EGFP) were designed, validated, and synthesized by Hanheng Biotech Corp. (Shanghai, China).

### Electrophysiology

Electrophysiology was performed as described in the references [37]. Briefly, mice were sacrificed after inhaling isoflurane and the brains were rapidly removed, iced, and cut into 400 μm slices with a vibratome (Leica VP1200S, Leica Microsystems Inc.). Slices were then incubated at 32 °C for 1 h in artificial CSF containing 3.5 mM KCl, 120 mM NaCl, 1.3 mM MgSO4, 10 mM D-Glucose, 1.25 mM NaH2PO4, 26 mM NaHCO3, 2.5 mM CACL2 (pH 7.4), ~ 300 mOsm, and bubbled with carbogen (95% O_2_ + 5% CO_2_). The slices were recovered at room temperature for at least 1 h before recording. We recorded field excitatory postsynaptic potentials (fEPSP) in the stratum radiatum of CA1 by stimulating the CA3 Schaeffer collateral / commissural axons. The baseline response was recorded every 20 s as the stimulus intensity that generated 30% of a maximum response. After 20 min of stable baseline recording, two columns of high-frequency stimulation (100 Hz, 1 s; at a 30-s interval) were performed to evoke LTP, which was continuously recorded for 60 min. Whole-cell recordings were performed on CA1 pyramidal neurons. Miniature excitatory postsynaptic current (mEPSC) signals were recorded at -70 mV in Artificial cerebrospinal fluid (ACSF) containing 0.5 μM tetrodotoxin. Miniature inhibitory postsynaptic current (mIPSC) signals were recorded at 0 mV in ACSF containing 0.5 μM tetrodotoxin.

### Morris water maze test

The Morris water maze (MWM) test was performed to assess the spatial learning and memory of mice according to a previous protocol with some modifications [38]. Briefly, in a dark circular pool (1.2 m in diameter and 0.5 m in height), a transparent round platform (7 cm in diameter) was placed in the center of the southeast corner. Before testing, the pool was filled with opacified water to a depth of 35 cm, 1.5 cm higher than the height of the platform, with the temperature of the water set at 22 ± 2 °C. Objects of different shapes and colors attached to the wall of each quadrant were used as landmarks. During the training period, each mouse underwent 4 trials daily for 5 consecutive days, each trial starting from a different location according to the semirandom sequence distribution decisions by Vorhees and Williams [38], with the mice facing the wall of the pool when placed into the water. The mice were allowed to swim freely for 60 s to locate the platform, and mice that failed to find the platform within 60 s were guided to the platform and allowed to stay there for 20 s. The latency was the average of the four trials. At 24 h after the last training trial, the memory retention test was conducted with the platform removed. Each mouse was given 60 s to explore the pool. The swimming performance of each mouse was recorded and analyzed with the Ethovision video tracking software (Noldus, Netherlands).

### RNA-sequencing analysis

Total RNA isolated from dorsal hippocampi was used for RNA-seq analysis. cDNA library construction and sequencing were performed by Gene Denovo Biotechnology Co. (Guangzhou, China). High-quality reads were aligned to the mouse reference genome using Bowtie2. Expression abundance and variations for each of the genes were normalized to fragments per kilobase of transcript per million mapped reads (FPKM) using RNA-seq by Expectation Maximization (RSEM). We identified differentially expressed genes (DEGs) between samples and performed clustering analysis and functional annotation. For genes, a fold change of ≥ 1.2 and a false discovery rate (FDR) of < 0.05 were considered as statistically significant. Pathways overrepresented by DEGs were annotated in the KEGG (Kyoto Encyclopedia of Genes and Genomes) database.

To identify groups of genes that correlated with ACSS2 upregulation in the 5 × FAD mice, we performed weighted gene coexpression network analysis (WGCNA) in R using the WGCNA package [39]. Signed hybrid coexpression networks were built for all WGCNAs. On the basis of the relationships between power and scale independence, the power of 5 was chosen for building scale-free topology. We used hybrid dynamic tree cutting, a minimum module size of 60 genes and mergeCutheight = 0.2. To assess the correlation of modules with treatment, we defined FAD-GFP as 0 and FAD-ACSS2 as 1. Modules significantly correlated with the treatment were annotated using R package anRichment (https://horvath.genetics.ucla.edu/html/CoexpressionNetwork/GeneAnnotation/index.html). Selected networks were visualized using VisANT [40].

### RT-qPCR

Total RNA extracted from the hippocampi or dorsal hippocampi with a Trizol reagent was subjected to cDNA synthesis with a Revert Aid First Strand cDNA Synthesis Kit (Thermo Fisher Scientific, K1622). Then, the cDNA was subjected to real-time quantitative PCR analysis with a SYBR Green Master (Roche, 04913914001) and specific primers. PCR primer sequences were shown in Appendix Table S3.

### Western blotting

Brain tissues were sonicated in a cold RIPA buffer (abcam, #ab156034) supplemented with protease inhibitor cocktail (Millipore, #539131), PMSF (CST, #8553), phosphatase inhibitor cocktail (MCE, #HY-K0022) and deacetylase inhibitors (5 mM Nicotinamide, 1 μM trichostatin A). The lysates were then centrifuged at 16,000 g for 25 min at 4 °C and the supernatants were collected. To prepare the cytoplasmic and nuclear fraction, the hippocampus newly collected was lysed using a Nuclear and Cytoplasmic Protein Extraction Kit (Beyotime Biotechnology, P0028) according to the manufacturer’s instructions. Afterwards, the protein in the lysates was measured with the BCA protein assay kit (Beyotime, cat#P0009) and adjusted to the same final concentration. The mixtures were heated at 100 °C for 10 min. Equal amounts of protein in the mixtures were separated by SDS-PAGE and transferred to PVDF membranes. The membranes were blocked in 5% bovine serum albumin (BSA) in tris-buffered saline containing 0.1% Tween-20 (TBST) at room temperature for one hour, followed by incubation with primary antibodies diluted in 5% BSA in TBST at 4 °C overnight. After three washes with TBST, the membranes were incubated with HRP-conjugated secondary antibody at room temperature for one hour. Immunoblot signals were detected using an ECL substrate. Quantification was conducted using the NIH ImageJ software. Applied antibodies were listed in Appendix Table S1.

### Immunofluorescence assays (IF)

Coronal brain sections through the dorsal hippocampus were cut on a freezing sliding microtome. Sections were washed and then blocked in TBS containing 0.3% Triton X-100, 1% BSA, and 10% normal donkey serum at room temperature for one hour, followed by incubation with primary antibodies diluted in TBS containing 0.3% Triton X-100, 1% bovine serum albumin (BSA) and 2.5% normal donkey serum at 4 °C overnight. After washing, the sections were incubated with a mixture of secondary antibodies at room temperature for one hour. Samples were then washed and counterstained with DAPI. Images were acquired under a Zeiss confocal microscope. The antibodies used were shown in Appendix Table S1.

### Immunohistochemistry assays (IHC)

The brains were sectioned coronally through the dorsal hippocampus on a freezing sliding microtome. Sections were washed and then treated with 3% hydrogen peroxide for 10 min. After washing, the sections were blocked in TBS containing 0.3% Triton X-100, 0.25% BSA, and 5% normal goat serum at room temperature for one hour, and subsequently incubated with primary antibodies diluted in TBS containing 0.3% Triton X-100, 0.25% BSA and 2% normal goat serum at 4 °C overnight. After washing, the sections were further incubated with a mixture of secondary biotinylated antibodies at room temperature for 90 min. Afterwards, the sections were rewashed and re-incubated with an ABC kit (Vector Labs, PK-6100) at room temperature for one hour. After another washing, immunoreactivity was detected using diaminobenzidine. Finally, the sections were thoroughly washed in TBST and mounted onto glass slides, air dried, dehydrated in a series of ethanol, and cleared in xylene. Images were obtained under an Olympus microscope. Applied antibodies were shown in Appendix Table S1.

### Acetyl-CoA ELISA

Acetyl-CoA levels in the hippocampus or dorsal hippocampus were measured using a mouse Ac-CoA ELISA Kit (Shanghai Yiyan Biotech Co. Ltd, EY12009-M) according to the manufacturer’s instructions.

### ChIP-qPCR

Hippocampi or dorsal hippocampi were removed quickly, snap frozen, and stored at -80 °C until further processing. Chromatin immunoprecipitation (ChIP) was performed using the Simple ChIP Plus Enzymatic Chromatin IP Kit (Magnetic Beads (Cell Signaling Technology, #9005) as described by the manufacturer with minor modifications. In brief, samples were finely minced and cross-linked with 1.5% formaldehyde for 10 min at room temperature and quenched by the addition of glycine for 5 min. Samples were then homogenized to create single cell suspension. Chromatin was sheared via incubation with 0.5 μl Micrococcal Nuclease at 37 °C for 20 min and then sonicated (a cycle of 100W, 1 s on/5 s off; 10 cycles) to break nuclear membrane. For immunoprecipitation, the diluted chromatin was incubated with 3 μl normal IgG (control), 3 μl ac-H4K12 (abcam, ab46983), or 3 μl ac-H3K9 (abcam, ab4441) antibodies overnight at 4 °C by constant rotation, followed by incubation with 30 μl of Protein G Magnetic Beads for an additional 2 h. Then, DNA was eluted from the beads and purified, and samples were subjected to RT-qPCR as described above using the indicated primer shown in Appendix Table S3. ChIP-qPCR results were calculated as the percentage of input DNA.

### Statistical analysis

All results are presented as means ± SEM, unless specified otherwise. GraphPad Prism 9.0 software was used for statistical analysis. The distribution of data was evaluated by the Shapiro**-**Wilk normality test and most of the data were normally distributed. The homogeneity of variances was assessed by Bartlett's test. The significance of differences was analyzed by unpaired two-tailed t-test or Mann**-**Whitney test, one-way or two-way analysis of variance (ANOVA) followed by post hoc Tukey’s multiple comparisons test, and, Scheirer-Ray-Hare test followed by the Dunn’s post-hot test. In addition, the escape latency was analyzed by the three-way ANOVA (Genotype × treatment × day), in which “day” was the repeated factor. The statistical parameters were specified in the figures and figure legends. A value of *P* < 0.05 was considered statistically significant.

## Results

### ACSS2 expression decreases in the cognition-related brain regions of middle-aged 5 × FAD mice and AD patients

**Fig. 1:**
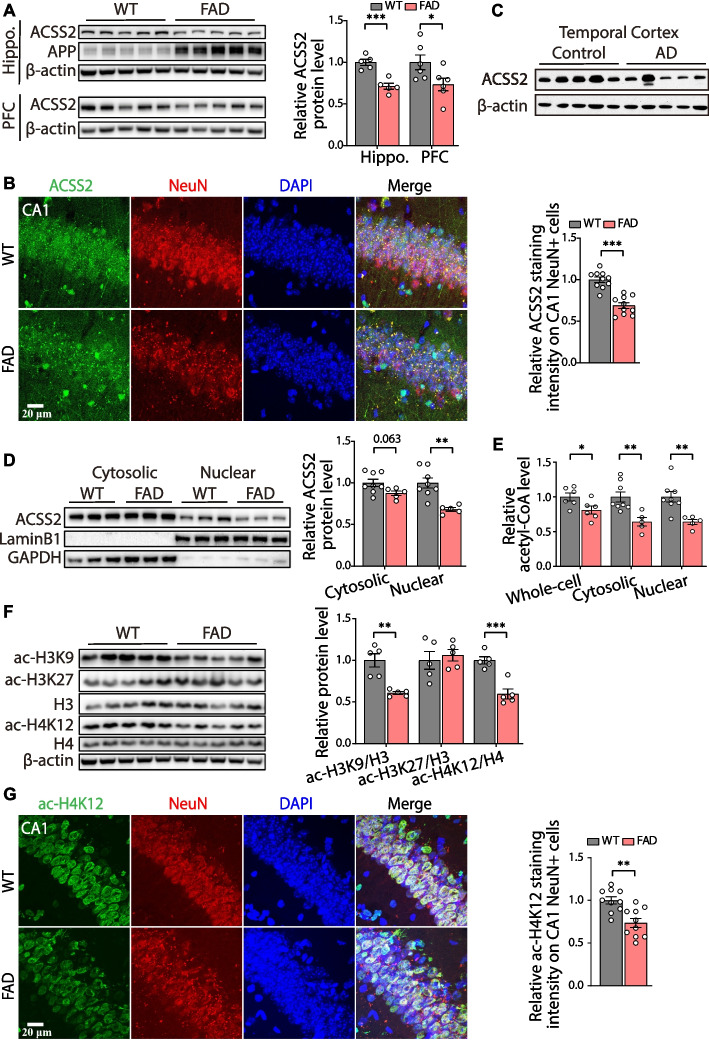
The decrease in ACSS2 expression and histone acetylation in the cognition-related brain regions of middle-aged 5 × FAD mice and AD patients. **A**, **B** Representative immunoblots and western blot analysis of ACSS2 in the hippocampus (hippo.) and prefrontal cortex (PFC) of the 10-month-old wild-type (WT) and 5 × FAD mice (hippocampus, *n* = 5 per group; prefrontal cortex, *n* = 6 per group) (**A**). Representative images of sections through the dorsal hippocampus immunostained with ACSS2 (green), NeuN (red), and DAPI (blue) in the 10-month-old WT and 5 × FAD mice (**B**, left panel), with the quantification of ACSS2 intensity on CA1 NeuN + cells shown in the right panel (*n* = 3 mice for WT and *n* = 3 mice for 5 × FAD) (**B**, right panel). **C** Western blotting analysis of ACSS2 in the temporal cortex of AD patients and the healthy individuals (*n* = 5 per group). **D** Representative immunoblots and western blot analysis of ACSS2 in the cytosolic and nuclear fractions of the hippocampus from the 10-month-old WT and 5 × FAD mice (*n* = 8 mice for WT and *n* = 5 mice for 5 × FAD). **E** Relative acetyl-CoA levels in the whole-cell, nucleus, and cytoplasm of the hippocampus from the 10-month-old WT and 5 × FAD mice (*n* = 5-8 per group). **F**, **G** Immunoblots and western blot analysis of ac-H3K9/H3, ac-H3K27/H3, and ac-H4K12/H4 in the hippocampus (*n* = 5 per group) (**F**). Representative images of sections through the dorsal hippocampus immunostained with ac-H4K12 (green), NeuN (red), and DAPI (blue) in the 10-month-old WT and 5 × FAD mice (**G**, left panel), with the quantification of ac-H4K12 intensity on CA1 NeuN^+^ cells shown in the right panel (*n* = 3 mice for WT and *n* = 3 mice for 5 × FAD) (**G**, right panel). Data are expressed as mean ± SEM. * *P* < 0.05, ** *P* < 0.01, *** *P* < 0.001 by the unpaired two-tailed t-test

Histone acetylation controls synaptic plasticity [18] and contributes to cognitive deterioration in AD [41, 42]. However, the underlying mechanism of histone acetylation dysregulation in AD remains poorly understood. We first quantified the mRNA levels of enzymes involved in regulating histone acetylation in the hippocampus of 2-, 5-, and 8- month 5 × FAD mice, which is a well-established transgenic model of AD [35, 43]. The results showed no significant change in the level of most enzymes (Fig. S1A). However, an apparent decrease in mRNA level of *Acss2* (*P* = 0.046) and a consistent decrease in ACSS2 protein expression (*P* = 0.033) (Fig. S1A and B) were evident at 8 months of age, with no similar trends detected at 2 or 5 months old. As ACSS2 acts as a key metabolic enzyme that regulates histone acetylation in neurons and affects the spatial memory in adult mice [20], it indicates that a reduction in ACSS2 may be significant in the context of AD. We further examined the ACSS2 levels in the 10-month-old 5 × FAD mice. Western blotting results showed a decline in the levels of ACSS2 in both the hippocampus (*P* < 0.001) and the prefrontal cortex (*P* = 0.046) of the 5 × FAD mice when compared with age-matched wildtype (WT) mice (Fig. 1A). We then performed the immunofluorescent co-staining of ACSS2 and NeuN (a marker of neurons) in hippocampal slices. The results showed that ACSS2 intensity in CA1 pyramidal neurons was markedly decreased in the 5 × FAD mice (*P* < 0.001) (Fig. 1B). We also found that ACSS2 protein level showed a downward trend in the temporal cortex from the post-mortem tissue of AD patients when compared with that of the healthy individuals (Fig. 1C). These results indicate that the expression of ACSS2 in the brain of patients with AD and 5 × FAD mice was significantly decreased.

### Histone acetylation decreases in the hippocampus of middle-aged 5 × FAD mice

ACSS2 is present in both cytosol and nucleus [44]. Western blotting of the cytosolic and nuclear fractions of hippocampal tissue demonstrated that nuclear ACSS2 was significantly attenuated in 10-month-old 5 × FAD mice when compared with age-matched WT mice (*P* = 0.002) (Fig. 1D). As ACSS2 is an acetyl-CoA synthetase that catalyzes the production of acetyl-CoA from acetate [45], we next examined the levels of acetyl-CoA in hippocampal whole-cell, cytosolic, and nuclear lysates. Colorimetric assays showed that the acetyl-CoA levels in whole-cell (*P* = 0.002), cytoplasm (*P* = 0.005), and nucleus (*P* = 0.004) were significantly lower in the 10-month-old 5 × FAD mice than in age-matched WT mice (Fig. 1E). As histone acetylation manipulated by nuclear acetyl-CoA homeostasis is tightly linked to neuronal gene expression [20], we next examined histone acetylation markers linked to learning and memory enhancement (ac-H3K9, ac-H3K27, and ac-H4K12) in the hippocampi of 2-, 5-, 8-, and 10-month-old 5 × FAD mice, respectively. Western blotting results showed that the levels of ac-H3K9 (*P* = 0.001) and ac-H4K12 (*P* < 0.001) were decreased in the 10-month-old 5 × FAD mice (Fig. 1F), while no reduction was found for mice at 2-, 5-, and 8-months old (Fig. S1B). Immunofluorescence staining also revealed that ac-H4K12 was markedly decreased in the CA1 pyramidal neurons of the 5 × FAD mice (*P* = 0.001) (Fig. 1G). These data suggest that the decrease of ACSS2 is accompanied by downregulation of acetyl-CoA and histone acetylation levels in middle-aged 5 × FAD mice.

### Age-dependent decline in ACSS2 expression is positively correlated with impaired spatial cognition in 5 × FAD mice

**Fig. 2:**
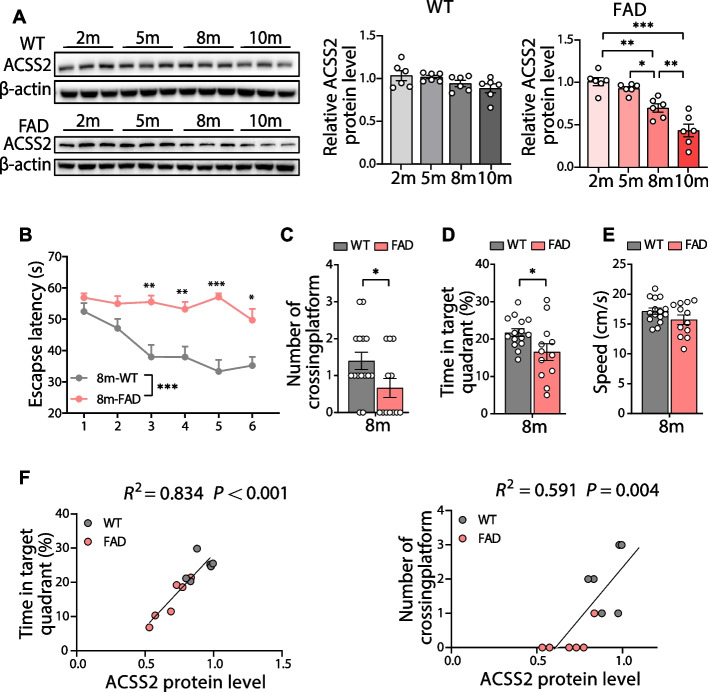
The decrease in hippocampal expression of ACSS2 is associated with cognitive impairment in middle-aged 5 × FAD mice. **A** Representative immunoblots and quantitative analyses of ACSS2 in the hippocampi of the 2-, 5-, 8- and 10-month-old WT or 5 × FAD mice (*n* = 6 per group). **B**-**E** The 8-month-old WT and 5 × FAD mice were examined by the Morris water maze (MWM) test. Escape latency (**B**) to the platform position during the training trials (1-5d) and the probe (6d) trial. The number of platform-position crossings (**C**), the percentage of time spent in the target quadrant (**D**), and the speed (**E**) in the probe (6d) trial. *n* = 15 and 12 mice for 8-month-old WT and 5 × FAD, respectively. **F** Linear regression demonstrated a positive correlation of ACSS2 protein level with the percentage of time spent in the target quadrant (**F**, left panel) or the number of platform-position crossings (**F**, right panel) in the MWM test (*n* = 6 mice/group). Data are expressed as mean ± SEM. Statistical significance was calculated by unpaired Mann**-**Whitney test (**C**), unpaired two-tailed t-test (**D**-**E**), linear regression analysis (**F**), one-way ANOVA (**A**), and two-way ANOVA (**B**) followed by the Tukey’s post-test. * *P* < 0.05, ** *P* < 0.01, *** *P* < 0.001

We further quantified hippocampal ACSS2 level during aging. The analyses revealed that ACSS2 protein levels in the 5 × FAD mice were markedly decreased at 8-months of age (8 m vs. 2 m, *P* = 0.002; 8 m vs. 5 m, *P* = 0.025) and aggravated strikingly at 10 months of age (10 m vs. 2 m, *P* < 0.001; 10 m vs. 8 m, *P* = 0.009) (Fig. 2A), while no significant changes were observed in WT mice. Next, Morris water maze (MWM) test was performed to assess the spatial learning and memory of the 8-month-old 5 × FAD mice. The results showed that compared with age-matched WT mice, the 8-month-old 5 × FAD mice displayed a longer escape latency to the platform position during the training trials (1-5d) and the probe (6d) trial [genotype: F (1, 25) = 29.03, *P* < 0.001; time: F (4.388, 109.7) = 6.071, *P* < 0.001] (Fig. 2B). Moreover, during the test period, the number of platform crossings (*P* = 0.046) (Fig. 2C) and the time spent in the target quadrant (*P* = 0.034) (Fig. 2D) decreased in the 5 × FAD mice. These results suggest that deficits in spatial learning and memory are evident in the 5 × FAD mice at 8-months of age. However, no similar results were observed in the 5 × FAD mice at 5**-**6 months of age in our laboratory. More importantly, further analyses revealed a significant positive correlation of the ACSS2 protein level with the percentage of time spent in the target quadrant (*R*^*2*^ = 0.834, *P* < 0.001) or the number of platform-position crossings (*R*^*2*^ = 0.591, *P* = 0.004) in the MWM test (Fig. 2F). These results suggest that age-associated attenuation in hippocampal expression of ACSS2 is directly related to spatial cognitive decline in the 5 × FAD mice.

### The ACSS2 upregulation alleviates the cognitive impairment in middle-aged 5 × FAD mice

To assess the role of ACSS2 in hippocampus-mediated spatial learning and memory, we downregulated ACSS2 levels in the dorsal hippocampus of 8-month-old WT mice and age-matched 5 × FAD mice via the injection of adeno-associated shAcss2 virus (Fig. S2A-C). After five weeks, the effect of *Acss2* knockdown on the spatial learning and memory was evaluated by the MWM task. Compared with the WT-GFP mice, the WT-shAcss2 mice displayed a longer escape latency to the platform during the training trials (1-5d) and the probe (6d) trial [genotype: F (1, 78) = 62.31, *P* < 0.001; time: F (5, 78) = 5.141, *P* < 0.001; *Acss2* knockdown: F (1, 78) = 14.11, *P* < 0.001] (Fig. S2D). During the probe trial, the WT-shAcss2 mice initiated significantly fewer target entries (*P* = 0.027) (Fig. S2E) and spent markedly less time in the target quadrant (*P* = 0.037) (Fig. S2F), but with no noticeable changes in swimming speed (Fig. S2G). These data are consistent with a recent report arguing that ACSS2 is essential for maintaining spatial learning and memory in adult mice [20]. Moreover, compared with the FAD-GFP mice, the FAD-shAcss2 mice also reported fewer target entries and less time spent in the target quadrant, though not statistically significant (Fig. S2E and F).

**Fig. 3:**
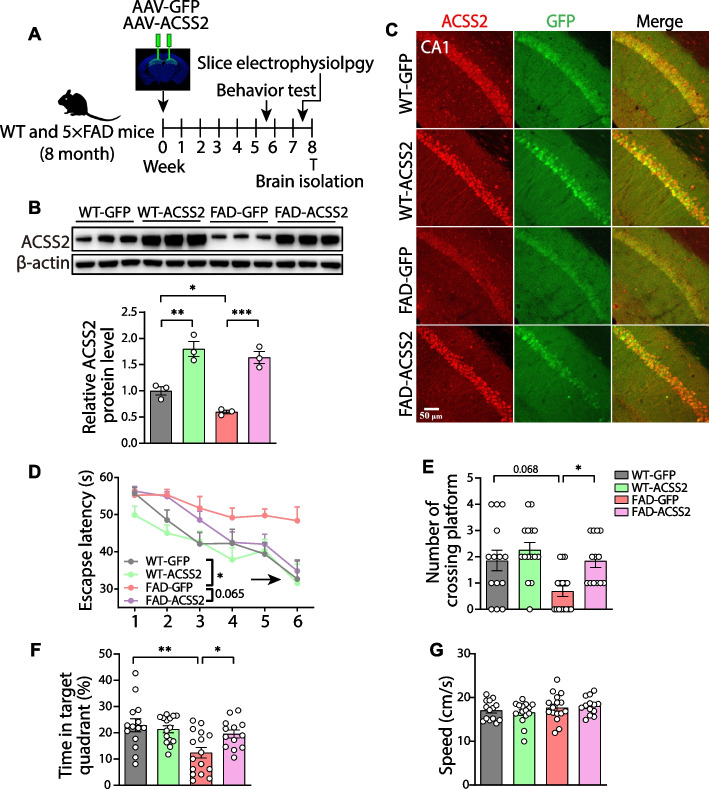
The improved cognitive performance by ACSS2 upregulation in middle-aged 5 × FAD mice. **A** The time schedule of the experimental procedure in up-regulating ACSS2. **B**, **C** Overexpression efficiency of *Acss2* was examined by western blot and immunofluorescence. Immunoblots and western blot analysis of ACSS2 in the dorsal hippocampus of mice injected with either GFP control virus or *Acss2* overexpression virus (*n* = 3 per group) (**B**). Representative images of ACSS2 (red) with GFP in the dorsal hippocampus of mice injected with either GFP control virus or *Acss2* overexpression virus (**C**). **D-G** *Acss2* overexpression mice were tested in the Morris water maze. Escape latency to the platform position during the training trails (1-5d) and the probe (6d) trial (**D**). The number of platform-position crossings (**E**), the percentage of time spent in the target quadrant (**F**), and the speed (**G**) in the probe (6d) trial. *n* = 14, 15, 16, and 13 mice for WT-GFP, WT-ACSS2, FAD-GFP, and FAD-ACSS2, respectively. Data are expressed as mean ± SEM. Statistical significance was calculated by two-way ANOVA (**B**, **F**, **G**) and three-way ANOVA (**D**) followed by the Tukey’s post-test, and Scheirer-Ray-Hare test followed by the Dunn’s post-hot test (**E**). * *P* < 0.05, ** *P* < 0.01, *** *P* < 0.001

We also injected adeno-associated virus expressing *Acss2* in the dorsal hippocampus of the 8-month-old 5 × FAD mice (Fig. 3A). Western blot and immunofluorescence analyses showed that ACSS2 was obviously upregulated in both the 5 × FAD and WT mice (Fig. 3B and C). Next, we examined whether ACSS2 upregulation can rescue the impaired spatial learning and memory in the MWM test. The results revealed that compared with the FAD-GFP mice, the FAD-ACSS2 mice displayed a shorter escape latency to the platform position during the training trials (1-5d) and the probe (6d) trial [genotype: F (1, 90) = 26.99, *P* < 0.001; time: F (5, 90) = 15.30, *P* < 0.001; *Acss2* overexpression: F (1, 90) = 11.48, *P* = 0.001] (Fig. 3D), suggesting that up-regulating ACSS2 can improve spatial learning. Consistently, during the test period, up-regulating ACSS2 significantly increased the number of platform crossings (*P* = 0.044) (Fig. 3E) and time spent in the target quadrant (*P* = 0.049) (Fig. 3F), without affecting the swimming speed (Fig. 3G). These results indicate that ACSS2 upregulation alleviates the cognitive impairment in the middle-aged 5 × FAD mice. Together, these data demonstrate that ACSS2 levels in the dorsal hippocampus is essential not only for spatial learning and memory in adult mice, but also for cognitive performance improvement in the late-stage 5 × FAD mice.

### The ACSS2 upregulation enhances the synaptic plasticity-associated pathway in middle-aged 5 × FAD mice

**Fig. 4:**
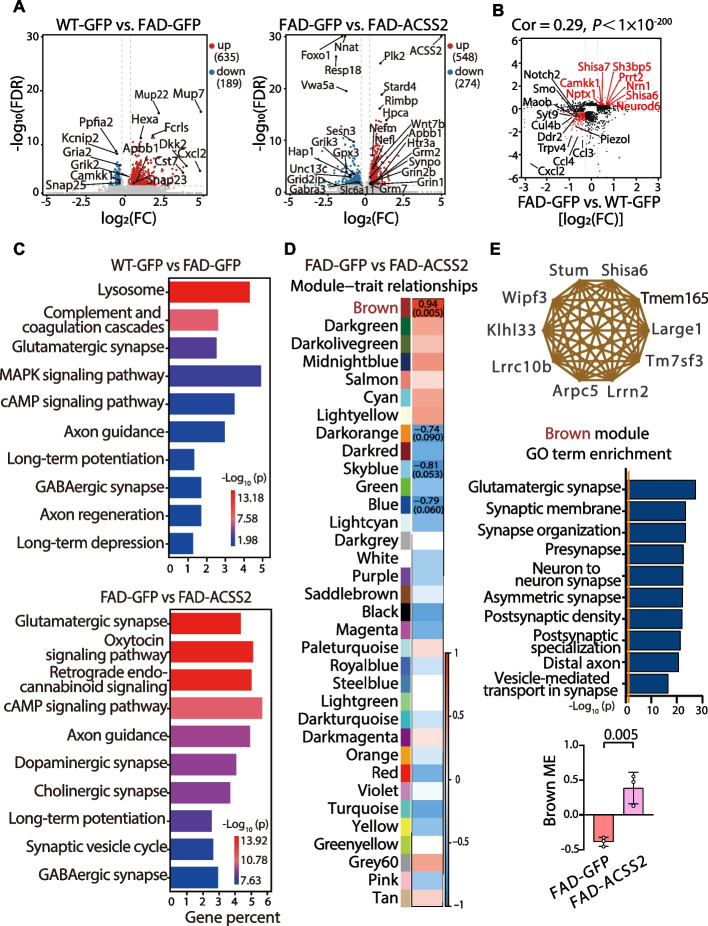
Transcriptomic profiling and WGCNA analysis of the ACSS2 upregulation in middle-aged 5 × FAD mice. **A**-**C** Transcriptomic profile of dorsal hippocampus from mice with ACSS2 upregulation. The volcano plot for differentially expressed genes (DEGs) (FDR < 0.05 and |fold change|≥ 1.2) in WT-GFP vs. FAD-GFP (**A**, left panel), FAD-GFP vs. FAD-ACSS2(**A**, right panel). Red plots represent upregulated DEGs. Blue plots represent downregulated DEGs. Gray points represent non-DEGs. *n* = 3 per group (**A**). Correlation analysis of fold change between WT-GFP vs. FAD-GFP and FAD-GFP vs. FAD-ACSS2 for genes with |fold change|≥ 1.2 (each gene corresponds to one point). The red dots denote genes that were downregulated (upregulated) in 5 × FAD mice which were upregulated (downregulated) by ACSS2 upregulation (**B**). KEGG pathway analysis for the DEGs between WT-GFP and FAD-GFP group, with the top 10 pathways shown (**C**, top panel). The top 10 pathways that were altered by ACSS2 upregulation in 5 × FAD mice (**C**, bottom panel). **D, E** WGCNA analysis of ACSS2 upregulation in 5 × FAD mice. Modules associated with the comparison of FAD-ACSS2 with FAD-GFP mice (*n* = 3 per group) (**D**). Numbers in the heatmap show the correlation coefficient. Modules with positive values (red) indicate an upregulation in FAD-ACSS2 vs. FAD-GFP mice; modules with negative values (blue) indicate a down-regulation. Network plot of the top 10 genes with the highest intramodular connectivity (hub genes) in the brown module (**E**, top panel). Gene ontology (GO) term enrichment of the brown module using 1086 module genes (**E**, middle panel). The orange line indicates the threshold of significance (*P* = 0.05). The trajectory of the module eigengenes (MEs) for each transcript in the brown module between FAD-ACSS2 and FAD-GFP mice (E, bottom panel). Data are expressed as mean ± SEM. Statistical significance was calculated by unpaired two-tailed t-test (**E**, bottom). ** *P* < 0.01

To profile potential transcriptional changes in the FAD-ACSS2 mice, we first performed bulk-RNA sequencing of the dorsal hippocampal tissues from the WT-GFP, FAD-GFP, and FAD-ACSS2 mice. The differentially expressed genes (DEGs) were defined by FDR < 0.05 and |fold change|≥ 1.2. We observed substantial transcriptional changes [FAD-GFP vs. WT-GFP mice: 824 DEGs (635 up-regulated and 189 down-regulated genes); FAD-ACSS2 vs. FAD-GFP mice: 822 DEGs (548 up-regulated and 274 down-regulated genes)] (Fig. 4A). Transcriptome-wide scatterplots showed a positive correlation in the gene-fold changes between FAD-GFP vs. WT-GFP mice and FAD-GFP vs. FAD-ACSS2 mice (Fig. 4B). To obtain mechanistic insights into the canonical pathways mediated by ACSS2, we performed Kyoto Encyclopedia of Genes and Genomes (KEGG) analysis for DEGs and found that synaptic plasticity-related signaling pathways, such as glutamatergic synapse, axon guidance, and long-term potentiation, were significantly altered in the 5 × FAD mice, which was also replicated in the FAD-ACSS2 mice (Fig. 4C).

Next, to illuminate gene expression changes in a system-level framework, we performed a weighted gene co-expression network analysis (WGCNA). We identified 34 modules between the FAD-GFP group and the FAD-ACSS2 group (Fig. 4D), in which one upregulated module was significantly correlated with ACSS2 upregulation, indicated as brown [*P* = 0.005, Fig. 4D and Fig. 4E (bottom panel)]. The enrichment analysis revealed that genes within the brown module were implicated in pathways related to “glutamatergic synapse”, “synaptic membrane”, “synapse organization”, “neuron to neuron synapse”, “asymmetric synapse”, “postsynaptic density”, and “postsynaptic specialization” (Fig. 4E, middle panel). These results indicate that ACSS2 upregulation promotes synaptic plasticity-related signaling pathways in the middle-aged 5 × FAD mice.

### The ACSS2 upregulation restores the level of glutamate receptors by increasing histone acetylation in the hippocampus of middle-aged 5 × FAD mice

**Fig. 5:**
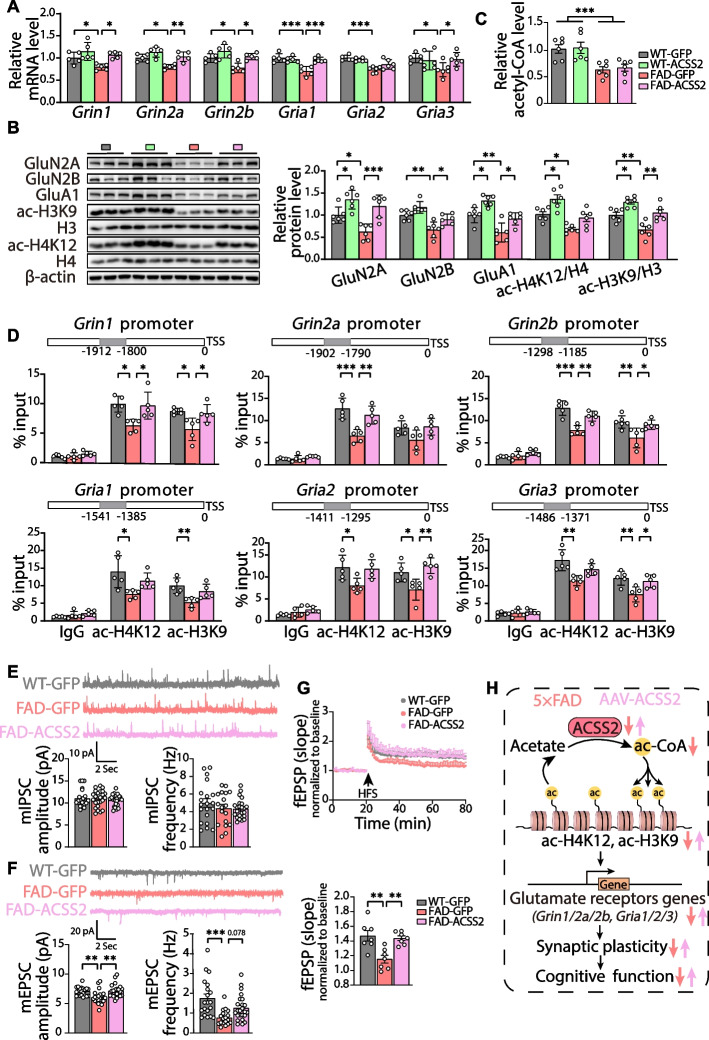
The ACSS2 upregulation-restored synaptic plasticity via the histone acetylation-mediated expression of glutamate receptors in middle-aged 5 × FAD mice. **A**, **B** The real-time quantitative PCR (RT-qPCR) analysis of genes encoding NMDA receptors subunits (*Grin1, Grin2a, Grin2b*) and AMPA receptors subunits (*Gria1, Gria2, Gria3*) in the dorsal hippocampi of the WT-GFP, WT-ACSS2, FAD-GFP, and FAD-ACSS2 mice (**A**). *n* = 5 per group. Representative immunoblots and quantitative analyses of NMDA receptors subunits (GluN2A, GluN2B), AMPA receptors subunits (GluA1), ac-H3K9/H3, and ac-H4K12/H4 in the dorsal hippocampus (**B**). *n* = 6 per group. **C** Relative acetyl-CoA levels in the dorsal hippocampus (*n* = 6 per group). **D** ChIP-qPCR analyses of the enrichment of ac-H4K12 and ac-H3K9 at *Grin1, Grin2a, Grin2b, Gria1, Gria2*, and *Gria3* promoters in the dorsal hippocampi of the WT-GFP, FAD-GFP, and FAD-ACSS2 mice. Rabbit IgG was used as the negative control. The bars on the top represented the location of primers for detecting ac-H4K12 and ac-H3K9 occupancy. TSS, transcriptional start site. *n* = 5 per group. **E** Representative traces of mIPSCs. mIPSCs were recorded at a holding potential of 0 mV (scale bar = 100 ms, 10 pA) (E, top panel). Quantitative analysis of the amplitude and frequency of mIPSCs (**E**, bottom panel). *n* = 23–24 cells per group. **F **Representative traces of mEPSCs. mEPSCs were recorded at a holding potential of –70 mV (scale bar = 100 ms, 10 pA) (**F**, top panel). Quantitative analysis of the amplitude and frequency of mEPSCs (**F**, bottom panel). *n* = 20–22 cells per group. **G** Hippocampal CA1 LTP recordings from the WT-GFP, FAD-GFP, and FAD-ACSS2 mice. Representative fEPSPs showed superimposed traces recorded during baseline and 60 min post-HFS in hippocampal CA1 area (**G**, top panel). HFS, high-frequency stimulation. fEPSP amplitude quantification during the last 10 min of LTP recording (**G**, bottom panel). *n* = 7 slices per group. **H** A proposed working model by which ACSS2 regulates synaptic plasticity and cognitive function in 5 × FAD mice. Data are expressed as mean ± SEM. Statistical significance was calculated by two-way ANOVA (**A**-**C**) and one-way ANOVA (**D**-**G**) followed by the Tukey’s post-test. * *P* < 0.05, ** *P* < 0.01, *** *P* < 0.001

The above transcriptome analysis strongly suggests that the glutamatergic synapse pathway was moderated by ACSS2 upregulation in the 5 × FAD mice. NMDA receptors (NMDARs) and AMPA receptors (AMPARs) mediate the induction and maintenance of Long-term potentiation (LTP) and Long-term depression (LTD), which is critical for synaptic plasticity and memory [46, 47]. Abnormal NMDAR and AMPAR functions are evident in AD [48]. Therefore, we performed RT-qPCR analysis to determinne mRNA levels of NMDAR subunits (*Grin1/2a/2b*) and AMPAR subunits (*Gria1/2/3*) in the dorsal hippocampus. Compared with WT mice, the 5 × FAD mice (9–10 months old) exhibited a statistically reduction in mRNA levels of *Grin1* (*P* = 0.016), *Grin2a* (*P* = 0.017), *Grin2b* (*P* = 0.028), *Gria1* (*P* < 0.001), *Gria2* (*P* < 0.001), and *Gria3* (*P* = 0.015), which were all increased by ACSS2 upregulation (*Grin1*, *P* = 0.022; *Grin2a*, *P* = 0.006; *Grin2b*, *P* = 0.011; *Gria1*, *P* < 0.001; *Gria2*, *P* = 0.063; *Gria3*, *P* = 0.011) (Fig. 5A). Consistent with the qPCR analysis, quantitative immunoblotting experiments also demonstrated a similar recovery of glutamate receptor proteins in the FAD-ACSS2 mice (GluN2A, *P* < 0.001; GluN2B, *P* = 0.037; GluA1, *P* = 0.020) (Fig. 5B). Together, these data indicate that the transcription and expression of glutamate receptors are statistically diminished in the hippocampus of the middle-aged 5 × FAD mice, which can be rescued by ACSS2 upregulation.

ACSS2 is one of the important regulators of histone acetylation [49, 50], which plays a critical role in neuronal gene expression [20]. To test whether ACSS2 upregulation restores the expression of glutamate receptors in the 5 × FAD mice by increasing histone acetylation, we performed the following experiments. Firstly, we examined the acetylation of histones (H3K9 and H4K12) in the dorsal hippocampus by immunoblotting analysis. The results showed that the ac-H3K9/H3 and ac-H4K12/H4 levels of the ACSS2 upregulation group in both the WT mice and 5 × FAD mice were significantly higher than those in the GFP control group (ac-H3K9/H3: FAD-GFP vs. FAD-ACSS2, *P* = 0.003, WT-GFP vs. WT-ACSS2, *P* = 0.023; ac-H4K12/H4: FAD-GFP vs. FAD-ACSS2, *P* = 0.090, WT-GFP vs. WT- ACSS2, *P* = 0.013) (Fig. 5B). Then, we detected the acetyl-CoA level in the dorsal hippocampus and found no difference between GFP control groups and ACSS2 upregulation groups, regardless of the genotype (Fig. 5C). Finally, we designed primers against the promoter regions of target genes (*Grin1/2a/2b* and *Gria1/2/3*) and measured the occupancy of ac-H3K9 and ac-H4K12 at these regions by ChIP-qPCR assays. The analyses revealed that the enrichment of ac-H4K12 at *Grin1* (*P* = 0.012), *Grin2a* (*P* < 0.001), *Grin2b* (*P* < 0.001), *Gria1* (*P* = 0.015), *Gria2* (*P* = 0.035), *Gria3* (*P* = 0.003) and that of ac-H3K9 at *Grin1* (*P* = 0.015), *Grin2a* (*P* = 0.103), *Grin2b* (*P* = 0.007), *Gria1* (*P* = 0.005), *Gria2* (*P* = 0.030), and *Gria3* (*P* = 0.008) were significantly downregulated in the dorsal hippocampus of the 5 × FAD mice (9**-**10 months old), which was reversed by ACSS2 upregulation (ac-H4K12: *Grin1*, *P* = 0.019; *Grin2a*, *P* = 0.007; *Grin2b*, *P* = 0.006; *Gria1*, *P* = 0.158; *Gria2*, *P* = 0.053; *Gria3*, *P* = 0.088; ac-H3K9: *Grin1*, *P* = 0.030; *Grin2a*, *P* = 0.069; *Grin2b*, *P* = 0.029; *Gria1*, *P* = 0.060; *Gria2*, *P* = 0.004; *Gria3*, *P* = 0.031) (Fig. 5D). These results suggest that ACSS2 upregulation enriches acetylated histones on the promoters and thus enhances the transcription of NMDA receptors and AMPA receptors in middle-aged 5 × FAD mice.

### The ACSS2 upregulation promotes synaptic plasticity by enhancing excitatory synaptic transmission in middle-aged 5 × FAD mice

As the loss of glutamate receptor expression in the 5 × FAD mice can result in diminished synaptic transmission [51], we performed whole-cell recordings with CA1 pyramidal cells in hippocampal slices from the WT-GFP, FAD-GFP, and FAD-ACSS2 mice, so as to determine any alterations in the basal synaptic function. No differences were detected in mIPSC frequency or amplitude among these groups (Fig. 5E). ACSS2 upregulation increased the amplitude and frequency of the downregulated mEPSC (*P* = 0.002;* P* = 0.078, respectively) in the 5 × FAD mice (Fig. 5F). The observed enhancement in mEPSC amplitude and frequency was consistent with the upregulated expression of AMPA and NMDA receptors, indicating that ACSS2 upregulation promotes excitatory synaptic transmission. Furthermore, to test the evoked transmission, we measured the long-term potentiation (LTP) recording from stratum radiatum in CA1 by stimulating hippocampal Schaffer collaterals. We found that the field excitatory postsynaptic potentials (fEPSPs) were obviously compromised in the 5 × FAD mice (*P* = 0.002), which was rescued by ACSS2 upregulation (*P* = 0.005) (Fig. 5G). Taken together, ACSS2 upregulation restores the synaptic plasticity by mediating the expression of NMDA- and AMPA- receptors via histone acetylation (H3K9 and H4K12) (Fig. 5H).

In addition to the glutamate system and electrophysiological characteristics, we examined the effect of ACSS2 upregulation on the pathological phenotype of AD, including Amyloid beta (Aβ) -deposition and gliosis [35, 52]. The Aβ protein level determined by western blotting analysis and Aβ plaque (6E10) content measured by immunohistochemical staining were prominent in the dorsal hippocampus of the FAD-GFP mice, but no significant differences were observed between the FAD-GFP and FAD-ACSS2 mice (Fig. S3A and B). Furthermore, markers of activated astrocytes (GFAP) and microglia (Iba1, CD68) were not different between the two groups (Fig. S3A and B). These results indicate that the rescuing effect of ACSS2 upregulation on synaptic function was independent of the typical AD pathology.

### Acetate supplementation upregulates the expression of glutamate receptors by increasing histone acetylation in the hippocampus of middle-aged 5 × FAD mice

**Fig. 6:**
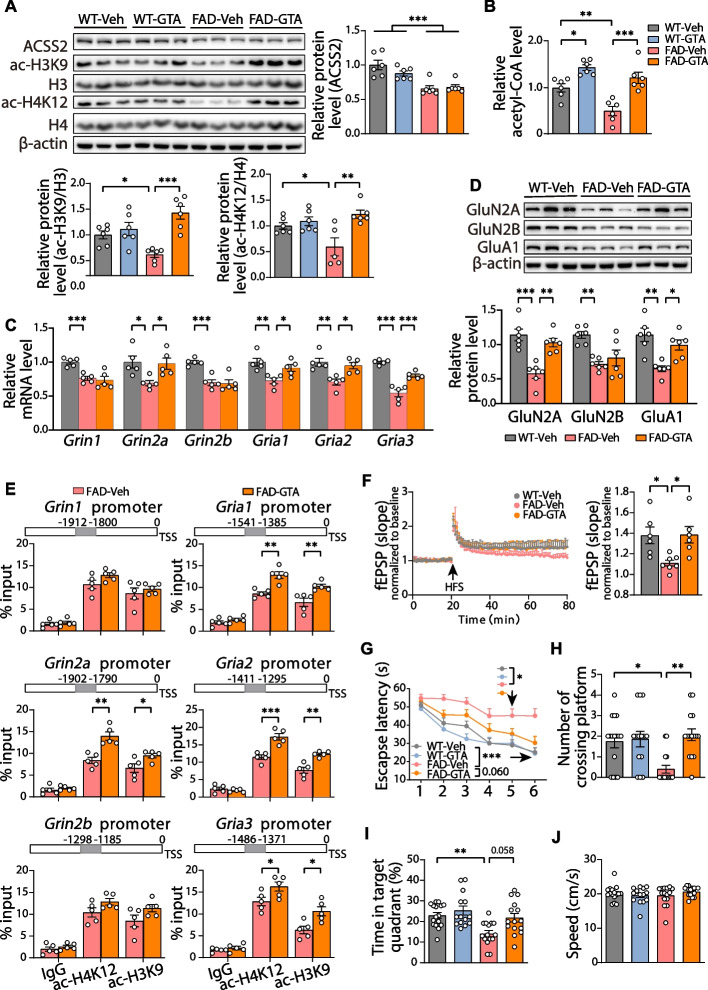
The improved synaptic plasticity and cognitive function by the supplementation of ACSS2 substrate via the histone acetylation-mediated upregulation of glutamate receptors in middle-aged 5 × FAD mice. **A** Representative immunoblots and quantitative analyses of ACSS2, ac-H3K9/H3, and ac-H4K12/H4 in the hippocampus. *n* = 6 per group. **B** Relative level of acetyl CoA in the hippocampal lysates of the WT-Veh, WT-GTA, FAD-Veh, and FAD-GTA mice. *n* = 6 per group. **C**, **D** The mRNA levels of genes encoding NMDA receptors (*Grin1, Grin2a, Grin2b*) and AMPA receptors (*Gria1, Gria2, Gria3*) in the hippocampi of the WT-Veh, FAD-Veh, and FAD-GTA mice (**C**). *n* = 5 per group. Representative immunoblots and quantitative analyses of NMDA receptors (GluN2A, GluN2B) and AMPA receptors (GluA1) in the hippocampus (**D**). *n* = 6 per group. **E** ChIP-qPCR analyses of the enrichment of ac-H4K12 and ac-H3K9 at *Grin1, Grin2a, Grin2b, Gria1, Gria2*, and *Gria3* promoters in the dorsal hippocampi of the FAD-Veh and FAD-GTA mice. Rabbit IgG was used as a negative control. The bars on the top represented the location of primers for detecting ac-H4K12 and ac-H3K9 occupancy. TSS, transcriptional start site. *n* = 5 per group. **F** Hippocampal CA1 LTP recordings from the WT-Veh, FAD-Veh, and FAD-GTA mice. Representative fEPSPs showed superimposed traces recorded during baseline and 60 min post-HFS in the hippocampal CA1 area (**F**, left panel). HFS, high-frequency stimulation. fEPSP amplitude quantification during the last 10 min of LTP recording (**F**, right panel). *n* = 7 slices per group. **G**-**J** GTA-treated mice were tested in the Morris water maze. Escape latency to the platform position during the training trails (1-5d) and the probe (6d) trial (**G**). The number of platform-position crossings (**H**), the percentage of time spent in the target quadrant (**I**), and the speed (**J**) in the probe (6d) trial. *n* = 16, 14, 15, and 15 mice for WT-Veh, WT-GTA, FAD-Veh, and FAD-GTA, respectively. Data are expressed as mean ± SEM. Statistical significance was calculated by three-way ANOVA (**G**), two-way ANOVA (**A**, **B**), one-way ANOVA (**C**, **D**, **F**) followed by the Tukey’s post-test, Scheirer-Ray-Hare test followed by the Dunn’s post-hot test (**H**-**J**), and unpaired two-tailed t-test (**E**). * *P* < 0.05, ** *P* < 0.01, *** *P* < 0.001

The above studies suggest that ACSS2 upregulation restored synaptic plasticity by increasing histone acetylation in the aged 5 × FAD mice. To verify whether replenishing ACSS2 substrate (acetate) can achieve similar protection against AD, we performed an intragastric administration of glyceryl triacetate (GTA, an FDA-approved food additive, 2 g/kg/day) to the 8-month-old 5 × FAD mice or WT mice every day for 1 month, and analyzed their brains for AD-related pathology. Compared with the FAD-Veh mice, the FAD-GTA mice showed a significantly higher level of both ac-H3K9/H3 (*P* < 0.001) and ac-H4K12/H4 (*P* = 0.002) (Fig. 6A). However, regardless of the genotypes, no significant difference in the ACSS2 protein level was evident between the GTA-treated groups and the Veh-treated groups (Fig. 6A). Unsurprisingly, the acetyl-CoA content in the hippocampus of either the WT-GTA group (1.44 times, *P* = 0.019) or the FAD-GTA group (2.46 times, *P* < 0.001) was higher than that of the genotype-matched groups (Fig. 6B). These results indicate that GTA supplementation upregulates the acetylation of histones by enhancing the Ac-CoA level in aged 5 × FAD mice.

Next, we measured the mRNA levels of NMDARs subunits (*Grin1/2a/2b*) and AMPARs subunits (*Gria1/2/3*) by RT-qPCR analysis. Compared with the FAD-Veh mice, the FAD-GTA mice reported significantly higher mRNA levels of *Grin2a* (*P* = 0.042), *Gria1* (*P* = 0.047), *Gria2* (*P* = 0.012), and *Gria3* (*P* < 0.001), though no significant difference in *GRIN1* (*P* = 0.899) and *Grin2b* (*P* = 0.823) was evident (Fig. 6C). Additionally, western blotting also displayed similar differences between the two groups (GluN2A, *P* = 0.001; GluN2B, *P* = 0.684; GluA1, *P* = 0.015) (Fig. 6D). ChIP-qPCR analysis was further performed to evaluate the enrichment of ac-H3K9 and ac-H4K12 on the promoters of NMDA receptors and AMPA receptors in the FAD-Veh and FAD-GTA mice. Compared with the FAD-Veh mice, the FAD-GTA mice reported increased occupancy of ac-H4K12 at *Grin2a* (*P* = 0.002), *Gria1* (*P* = 0.0012), *Gria2* (*P* < 0.001), and *Gria3* (*P* = 0.043), and enhanced enrichment of ac-H3K9 at *Grin2a* (*P* = 0.034), *Grin2b* (*P* = 0.008), *Gria2* (*P* = 0.001), *Gria3* (*P* = 0.013) (Fig. 6E), which were consistent with the upregulated expression of those genes. Collectively, these data demonstrate that supplementation of ACSS2 substrate upregulates the expression of glutamate receptors by increasing histone acetylation in the aged 5 × FAD mice.

### Acetate supplementation restores the synaptic plasticity and alleviates the cognitive impairment in middle-aged 5 × FAD mice

To investigate the effect of acetate supplementation on activity-dependent synaptic plasticity in the hippocampus of the 5 × FAD mice, we examined the long-term potentiation (LTP) recording from the stratum radiatum in CA1 by stimulating hippocampal Schaffer collaterals and observed that the marked LTP impairment in the 5 × FAD mice was significantly attenuated by acetate supplementation (*P* = 0.021) (Fig. 6F). Finally, we performed the MWM test to examine the therapeutic potential of acetate supplementation in memory tasks in the aged 5 × FAD mice. Compared with the FAD-Veh mice, the FAD-GTA mice tended to spend less time in reaching the platform during the training trials (1-5d) and the probe (6d) trial [genotype: F (1, 90) = 72.11, *P* < 0.001; time: F (5, 90) = 19.33, *P* < 0.001; GTA treatment: F (1, 90) = 16.94, *P* < 0.001] (Fig. 6G), suggesting the benefit of acetate supplementation to spatial learning in the 5 × FAD mice. During the probe trial, the FAD-GTA mice reported more target entries (*P* = 0.002) and more time in the target quadrant (*P* = 0.058), but with no difference in swimming speed (Fig. 6H-J). Taken together, these results suggest that supplementing ACSS2 substrate alleviates the cognitive impairment in aged 5 × FAD mice.

### Acss2 knockdown prevents the acetate supplementation-induced upregulation of glutamate receptors and cognitive improvement in middle-aged 5 × FAD mice

**Fig. 7:**
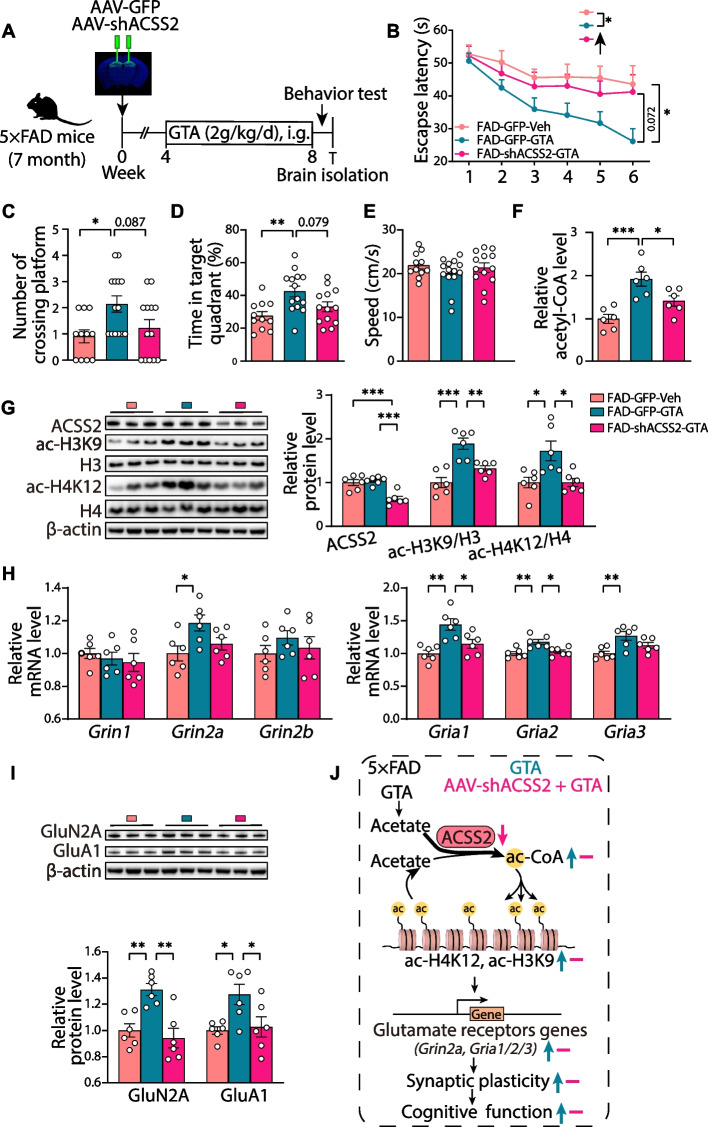
The prevention of acetate supplementation-induced upregulation of glutamate receptors and cognitive improvement by *Acss2* knockdown in middle-aged 5 × FAD mice. **A** The time schedule for the GTA treatment on the premise of *Acss2* knockdown in the dorsal hippocampus. **B**-**E** Mice were tested in the Morris water maze. Escape latency to the platform position during the training trails (1-5d) and the probe (6d) trial (**B**). The number of platform-position crossings (**C**), the percentage of time spent in the target quadrant (**D**), and the speed (**E**) in the probe (6d) trial. *n* = 11, 14, and 13 mice for FAD-GFP-Veh, FAD-GFP-GTA, and FAD-shAcss2-GTA, respectively. **F** Relative level of acetyl CoA in the dorsal hippocampi of the FAD-GFP-Veh, FAD-GFP-GTA, FAD-shAcss2-GTA mice. *n* = 6 per group. **G** Representative immunoblots and quantitative analyses of ACSS2, ac-H3K9/H3, and ac-H4K12/H4 in the dorsal hippocampus. *n* = 6 per group. **H**, **I** The mRNA levels of genes encoding NMDA receptors (*Grin1, Grin2a, Grin2b*) (H, left panel) and AMPA receptors (*Gria1, Gria2, Gria3*) (**H**, right panel) in the dorsal hippocampus of the FAD-GFP-Veh, FAD-GFP-GTA, FAD-shAcss2-GTA mice. *n* = 6 per group. Representative immunoblots and quantitative analyses of GluN2A and GluA1 in the dorsal hippocampus (**I**). *n* = 6 per group. **J** A proposed working model of acetate supplementation for inducing upregulation of glutamate receptors and cognitive improvement in middle-aged 5 × FAD mice, which was prevented by *Acss2* knockdown. Data are expressed as mean ± SEM. Statistical significance was calculated by two-way ANOVA (**B**) and one-way ANOVA (**C**-**I**) followed by the Tukey’s post-test. * *P* < 0.05, ** *P* < 0.01, *** *P* < 0.001

To further explore the role of ACSS2 in acetate-mediated cognitive protection in the aged 5 × FAD mice, we reduced ACSS2 expression in the dorsal hippocampus of the 7-month-old 5 × FAD mice by injecting adeno-associated shAcss2 virus. One month after viral transduction, mice were treated with GTA (2 g/kg/day, i.g.) for 4 weeks (Fig. 7A). During the MWM test (Fig. 7B-E), compared with the FAD-GFP-GTA mice, the FAD-shAcss2-GTA mice reported a longer latency to the platform position during the training trials (1-5d) and the probe (6d) trial, which was consistent with that of the FAD-GFP-Veh mice [time: F (4.048, 141.7) = 8.708, *P* < 0.001; treatment: F (2, 35) = 5.852, *P* = 0.006] (Fig. 7B). Similarly, compared with the FAD-GFP-GTA mice, the FAD-shAcss2-GTA mice exhibited fewer target entries (*P* = 0.087) and spent less time in the target quadrant (*P* = 0.079), with no significant differences in swimming speed found between the FAD-GFP-Veh mice and FAD-shAcss2-GTA mice (Fig. 7C-E). Thus, the MWM results suggest that *Acss2* knockdown compromises the benefit of acetate supplementation in improving spatial learning and memory in the 5 × FAD mice.

Next, we examined the levels of acetyl-CoA in the dorsal hippocampus of the 5 × FAD mice and found that the GTA-induced increase in acetyl-CoA content (*P* < 0.001) was inhibited by ACSS2 downregulation (*P* = 0.035) (Fig. 7F). Furthermore, the GTA treatment-induced upregulated levels of ac-H3K9/H3 (*P* < 0.001) and ac-H4K12/H4 (*P* = 0.013) were suppressed by *Acss2* knockdown (ac-H3K9/H3, *P* = 0.005; ac-H4K12/H4, *P* = 0.014) (Fig. 7G). These data indicate that acetate supplementation upregulates the levels of acetyl-CoA and acetylated histones in an ACSS2-dependent manner. Finally, RT-qPCR and western blot analysis revealed that *Acss2* knockdown restrained the GTA-mediated promotion of NMDARs (*Grin2a*/GluN2A) and AMPARs (*Gria1/*GluA1, *Gria2*, *Gria3*) expression in the dorsal hippocampus (Fig. 7H and I). Taken together, these findings demonstrate that GTA supplementation improves synaptic plasticity and cognitive function via the histone acetylation-mediated upregulation of glutamate receptors in aged 5 × FAD mice, which can be occluded by *Acss2* knockdown (Fig. 7J).

## Discussion

In the present study, reduced ACSS2 levels were observed in the temporal cortex of post-mortem tissues from AD patients and in the hippocampus and prefrontal cortex of the late-staged 5 × FAD mice. In 5 × FAD mice, diminished ACSS2 expression was correlated with the downregulated transcription of glutamate receptors that was mediated by reduced histone acetylation. Consequently, the synaptic plasticity was significantly impaired, leading to defective spatial cognition. Of note, we showed that supplementing acetic acid precursor glyceryl triacetate (GTA), the substrate of ACSS2, restored histone acetylation and glutamate receptor expression, leading to improved synaptic plasticity and ameliorated cognitive impairment in late-stage 5 × FAD mice. These findings document the involvement of ACSS2 in histone acetylation to control the expression of glutamate receptors in the AD brain and evidence the promising therapeutic potential of ACSS2 targeting and acetate administration in treating patients with intermediate or advanced AD.

The level of histone acetylation, especially on the genes related to learning and memory, significantly decreases in AD patients [26, 53] and AD animal models [25, 28]. Although the treatment of AD mouse models with histone deacetylase (HDACs) inhibitors has yielded some promising results [25, 28, 54], relevant clinical trials have not been available due to the poor selectivity and obvious side effects of HDACs inhibitors [55]. So far, there is no effective clinical treatment that targets abnormal histone acetylation of AD. Except for histone acetyl transferase (HAT) and HDAC, histone acetylation is governed by nuclear acetyl-CoA pools generated, in part, from local acetate by ACSS2 [20, 49, 56, 57]. In the current study, the level of ACSS2 and histone acetylation decreased in the hippocampus of the 5 × FAD mice at 8**-**10 months of age, which was accompanied by impaired spatial cognitive function. Importantly, we found that upregulation of ACSS2 in the dorsal hippocampus restored histone acetylation and spatial cognition in the late-stage 5 × FAD mice. A recent study reveals that ACSS2 directly transfers acetate between histone lysine residues to facilitate rapid transcriptional induction [58], which lends strong support to our findings that upregulation of ACSS2 levels significantly improved histone acetylation, even though no change was found in the overall acetyl-CoA levels (Fig. 5C). Taken together, the above evidence suggests that ACSS2 is a key molecular pathological feature that is directly related to cognitive function in middle and late AD. Therefore, the development of ACSS2 agonists is expected to be one of the novel approaches for the treatment of AD.

Acetyl-CoA is a pivotal biomolecular hub for energy metabolism and histone acetylation, and its homeostasis is critical for spatial memory in mice [21, 24]. Under nutritionally sufficient conditions, acetyl-CoA is largely derived from glucose, especially in the brain, where a high demand for glucose is always imminent, while hypoxia and other nutritional challenges lead to the utilization of acetate as a major metabolic source-an adaptation which has been well studied particularly in the context of the tumor microenvironment, including glioma [59–64]. In the present study, we found that the level of acetyl-CoA decreased in the hippocampus of the late-stage 5 × FAD mice, especially in the nuclear extracts (Fig. 1E), which is consistent with previous reports of abnormal glucose metabolism in the brain of AD patients [65, 66] and AD mice [67]. We found that GTA supplementation (an FDA-approved food additive) increased the total acetyl-CoA and histone acetylation levels in the hippocampus of the late-stage AD mice (Fig. 6A-B), which was obviously prevented in the *Acss2*-knockdown mice (Fig. 7F-G). These results suggest that the utilization of acetate by ACSS2 is a protective and adaptive metabolic process in the AD brain. The bacterial metabolism of dietary fiber and alcohol consumption and hepatic metabolism of ethanol directly contribute to circulating acetate [68, 69], which echoes the epidemiological evidence that a Mediterranean diet [70–73] and light alcohol consumption [74] reduce the risk of developing AD. Therefore, exogenous acetate supplementation is expected to be an effective treatment for patients with advanced AD.

Glutamate receptors are required for the induction and maintenance of long-term potentiation (LTP), one form of synaptic plasticity [75, 76]. Extensive research has documented that synaptic transmission and plasticity are impaired in AD or Aβ-exposed wild-type hippocampus, which is dependent on NMDARs, AMPARs, and downstream pathways [51, 77–81]. Here, we found that the late-stage AD mice exhibited a decreased enrichment of ac-H3K9 and ac-H4K12 on the promoters of regions of glutamate receptors, suggesting that the loss of glutamate receptor transcription in AD may be caused by aberrant histone acetylation. Consistent with this, increasing ac-H3K9 and ac-H4K12 via ACSS2 upregulation (Fig. 5) or GTA supplementation (Fig. 6) restored the expression of glutamate receptors. More evidence of the importance of histone acetylation in regulating glutamate receptor expression can be further acquired from the previous findings in substance abuse models [82, 83] and in a Shank3 mouse model of autism [84]. Of note, no effects of ACSS2 upregulation on Aβ deposition and gliosis were observed in the 5 × FAD mice (Fig. S3), suggesting that the rescuing effect of ACSS2 on synaptic function is independent of the typical AD pathology. Given the direct correlation between synaptic plasticity and cognitive impairment in AD, therapeutic strategies built on “ACSS2/histone acetylation/glutamate receptor” will provide novel perspectives for the treatment of AD.

In addition to histone acetylation, histone methylation has also been extensively studied in normal cognition [85, 86] and AD brain [51]. In recent years, various histone acylation modifications have been discovered, such as propionylation, butyrylation, and succinylation [87]. More recently, histone lactylation (H4K12) has been shown to be enriched at the promoters of glycolytic genes, which exacerbates glucose metabolism disorder and leads to microglial dysfunction in AD [43]. Since ACSS2 is widely expressed in neuronal cells in the brain, further studies are needed to clarify the role of ACSS2 expression in astrocytes under the neuropathology of AD. In addition, only a single dose of GTA was used in this study, so the dosage effect on cognition needs to be further explored.

## Conclusions

Our study has revealed that ACSS2 downregulation mediates a reduction in glutamate receptor expression through histone acetylation, which exacerbates synaptic plasticity impairment in AD. These processes can be rescued by acetate supplementation, which may serve as a promising therapeutic strategy for AD treatment.

## Supplementary Information

**Additional file 1:** **Table S1. **Key source table. **Table S2. **Information About Human Samples. **Table S3. **List of primers used in this study.

**Additional file 2:** **Fig. S1 **The levels of enzymes involved in regulating histone acetylation and acetyl-CoA level in the hippocampi of 2-, 5- and 8-month-old 5×FAD mice. **A** The RT-qPCR analysis of *Acss1, Acss3, Acss2, Acly, Pdha1, Hats*, and *Hdacs*in the hippocampus of 2-, 5- and 8-month-old WT and 5×FAD mice.** B** Immunoblots and western blot analyses of ACSS2, ac-H3K9/H3, ac-H3K27/H3, ac-H4K12/H4, and Aβin the hippocampi of 2-, 5- and 8-month-old WT and 5×FAD mice. Data are expressed as mean ± SEM. * *P* < 0.05 by the unpaired two-tailed t-test.

**Additional file 3:** **Fig. S2. **The impaired spatial learning and memory in WT and 5×FAD mice by ACSS2 knockdown.** A** The time schedule of the experimental procedure of ACSS2 knockdown. **B, C** Knockdown efficiency of shAcss2 was examined by western blot and immunofluorescence. Immunoblots and western blot analysis of ACSS2 in the dorsal hippocampus of mice injected with either GFP control virus or ACSS2 knockdown virus. Representative images of ACSS2with GFP in the dorsal hippocampus from mice injected with either GFP control virus or ACSS2 knockdown virus. **D-G**ACSS2 knockdown mice were tested in the Morris water maze. Escape latency to the platform position during the training trailsand the probe trial. The number of platform-position crossings, the percentage of time spent in the target quadrant, and the speedin the probetrial. *n*= 12, 14, 13, and 12 mice for WT-GFP, WT-shAcss2, FAD-GFP, and FAD-shAcss2, respectively. Data are expressed as mean ± SEM. Statistical significance was calculated by two-way ANOVAand three-way ANOVAfollowed by the Tukey’s post-test, and by Scheirer-Ray-Hare test followed by the Dunn’s post-hot test. * *P* < 0.05, ** *P*< 0.01.

**Additional file 4:** **Fig. S3. **The minimal effect of the ACSS2 upregulation on Aβ pathology and gliosis in middle-aged 5×FAD mice.**A **Representative immunoblots and quantitative analyses of Aβ, GFAP, and Iba1in the dorsal hippocampi of the WT-GFP, WT-ACSS2, FAD-GFP, and FAD-ACSS2 mice. *n* = 3 per group.** B** Representative images of immunohistochemical staining for Aβ , GFAP, and CD68in the dorsal hippocampus. Data are expressed as mean ± SEM. Statistical significance was calculated by the unpaired two-tailed t-testand two-way ANOVA followed by the Tukey’s post-test. ** *P* < 0.01, *** *P* < 0.001.

**Additional file 5.**

## Data Availability

The RNA-seq data from this publication have been deposited to the Genome Sequence Archive (GSA) (http://bigd.big.ac.cn/gsa) under accession no. CRA007557.

## Abbreviations

5 × FAD: 5 Familial AD mutations
AAV: Adeno-associated virus
Aβ: Amyloid-β
ACLY: ATP-citrate lyase
ACSF: Artificial cerebrospinal fluid
ACSS2: Acyl-CoA synthetase short-chain family member 2
AD: Alzheimer’s disease
AMPARs: α-Amino-3-hydroxy-5-methyl-4-isoxazolepropionic acid receptors
ANOVA: Analysis of variance
APP: Amyloid precursor protein
ChIP: Chromatin immunoprecipitation
ChIP-qPCR: Chromatin immunoprecipitation-quantitative PCR
DEGs: Differentially expressed genes
ELISA: Enzyme-linked immunosorbent assay
FDR: False discovery rate
fEPSPs: Field excitatory postsynaptic potentials
FPKM: Fragments per kilobase of transcript per million mapped reads
GFAP: Glial fibrillary acidic protein
GFP: Green fluorescent protein
GluA1/2/3 (*Gria1/2/3*): Glutamate ionotropic receptor AMPA type subunit 1/2/3
GluN1/2A/2B (*Grin1/2a/2b*): Glutamate ionotropic receptor NMDA type subunit 1/2A/2B
GO: Gene ontology
GSA: Genome sequence archive
GTA: Glyceryl triacetate
HATs: Histone acetyltransferases
HDACs: Histone deacetylases
HRP: Horseradish peroxidase
Iba1: Ionized calcium binding adaptor molecule 1
IF: Immunofluorescence assays
IHC: Immunohistochemistry assays
KEGG: Kyoto encyclopedia of genes and genomes
LTD: Long-term depression
LTP: Long-term potentiation
MCI: Mild cognitive impairment
mEPSC: Miniature excitatory postsynaptic current
MEs: Module eigengenes
mIPSC: Miniature inhibitory postsynaptic current
MWM: Morris water maze
NMDARs: N-methyl-D-aspartate receptors
PDC: Pyruvate dehydrogenase complex
PFC: Prefrontal cortex
PS1: Presenilin 1
RNA-seq: Ribonucleic acid sequencing
RSEM: RNA-seq by expectation maximization
RT-qPCR: Real-time quantitative PCR
SEM: Standard error of the mean
TBS: Tris-buffered saline
Veh: Vehicle
WGCNA: Weighted gene co-expression network analysis
WT: Wild-type
