# Supplementary material for: ACSS2-dependent histone acetylation improves cognition in mouse model of Alzheimer’s disease
Source: Mol Neurodegener. 2023 Jul 12;18:47. doi: 10.1186/s13024-023-00625-4 (PMC10339567; doi:10.1186/s13024-023-00625-4)
Supplement: Supplementary file 1 — Additional file 1: Table S1. Key source table. Table S2. Information About Human Samples. Table S3. List of primers used in this study. [file 13024_2023_625_MOESM1_ESM.docx]

**Table S1. Key source table**

| **REAGENT or RESOURCE** | **SOURCE** | **IDENTIFIER** |
| --- | --- | --- |
| **Antibodies** |  |  |
| ACSS2 | Cell Signaling Technology | 3658 |
| APP | Millipore | 17160 |
| Aβ (6E10) | Biolegend | 803001 |
| GluN2A | Millipore | AB1555 |
| GluN2B | abcam | ab65783 |
| GluA1 | Millipore | AB1504 |
| ac-H3K27 | abcam | ab4729 |
| ac-H4K12 | abcam | ab46983 |
| ac-H3K9 | abcam | ab4441 |
| H3 | abcam | ab10799 |
| H4 | abcam | ab10158 |
| GFAP | SYSY | 173 011 |
| Iba1 | abcam | ab15690 |
| β-actin | abcam | ab8227 |
| Lamin B1 | abcam | ab16048 |
| GAPDH | abcam | ab9485 |
| NeuN | abcam | ab104224 |
| CD68 | abcam | ab53444 |
| Goat Anti-Mouse IgG H&L (HRP) | abcam | ab205719 |
| Goat Anti-Rabbit IgG H&L (HRP) | abcam | ab205718 |
| Alexa Fluor488-conjugated donkey anti-rabbit IgG | Invitrogen | A-21206 |
| Alexa Fluor594-conjugated donkey anti-rabbit IgG | Invitrogen | A-21207 |
| Alexa Fluor594-conjugated donkey anti-mouse IgG | Invitrogen | A-21203 |
| Goat Anti-Rabbit IgG Antibody (H+L), Biotinylated | Vector labs | BA-1000-1.5 |
| Goat Anti-Mouse IgG Antibody (H+L), Biotinylated | Vector labs | BA-9200-1.5 |
| Goat Anti-Rat IgG Antibody (H+L), Biotinylated | Vector labs | BA-9400-1.5 |
| **Biological samples** |  |  |
| Frozen postmortem brain tissues from patients with AD and healthy controls | National Human Brain Bank for Development and Function, Chinese Academy of Medical Sciences and Peking Union Medical College, Beijing, China | N/A |
| **Chemicals** |  |  |
| Glyceryl triacetate (GTA) | Sigma-Aldrich | 90240 |
| Trichostatin A | Sigma-Aldrich | T1952 |
| Nicotinamide | Sigma-Aldrich | V900517 |
| **Critical commercial assays** |  |  |
| SimpleChIP Plus Enzymatic Chromatin IP Kit (Magnetic Beads | Cell Signaling Technology | 9005 |
| Mouse Ac-CoA ELISA Kit | Shanghai Yiyan Biotech Co. Ltd | EY12009-M |
| **Viral vector** |  |  |
| AAV-ACSS2 (rAAV-CMV-ACSS2-HA-P2A-EGFP-WPRE-pA) and AAV-GFP (rAAV-CMV-HA-P2A-EGFP-WPRE-pA) | Shumi Brain Science and Technology Co., LTD | N/A |
| AAV-shACSS2 (pHBAAV2/9-U6-m-ACSS2 shRNA-CMV-EGFP) and AAV-GFP (pHBAAV2/9-U6-MCS-CMV-EGFP) | Hanheng Biotech Corp. | N/A |
| **Experimental models: Organisms/strains** |  |  |
| Mouse: 5×FAD | Inhouse breeding | N/A |
| **Oligonucleotides** |  |  |
| Primers for ChIP-qPCR, see Table S3 | This paper | N/A |
| Primers for RT-qPCR, see Table S3 | This paper | N/A |
| Critical commercial assays |  |  |
| **Software and algorithms** |  |  |
| Etho-vision video tracking software | Noldus | N/A |
| GraphPad Prism 9.0 | GraphPad software | <https://www.graphpad.com/> |
| ImageJ software | National Institutes of Health | https://imagej.nih.gov/ij/download.html |

**Table S2. Information About Human Samples**

| **Case** | **PMI (h)** | **Age (years)** | **Sex** | **Disease stage** |
| --- | --- | --- | --- | --- |
| Control 1 | 21.5 | 78 | Male | Braak 0 |
| Control 2 | 4.3 | 70 | Male | Braak 0 |
| Control 3 | 23.5 | 93 | Male | Braak 0 |
| Control 4 | 4 | 95 | Male | Braak 0 |
| Control 5 | 3 | 89 | Male | Braak 0 |
| AD 1 | 6 | 93 | Male | Braak Ⅰ |
| AD 2 | 9.5 | 94 | Male | Braak Ⅰ |
| AD 3 | 8.8 | 87 | Male | Braak Ⅰ |
| AD 4 | 13.5 | 78 | Male | Braak Ⅰ |
| AD 5 | 4 | 77 | Male | Braak Ⅰ |
| Postmortem interval (PMI) is presented in hours between time of patient death and necropsy. | | | | |

**Table S3. List of primers used in this study**

| **Gene** | **Forward primer sequence (5'→3')** | **Reverse primer sequence (5'→3')** |
| --- | --- | --- |
| *Acss2* | CCATTGCCACACCAGACTAC | TCAGCCACCGTAGATGTATCC |
| *Acss1* | CCACCAAGATCGCCAAGTATG | TTCCTCAGTAGTCTCCTCATCAC |
| *Acss3* | GGCTGCCTTGTTTACAGCAC | GAACGGGAACTCGGAAGACT |
| *Acly* | CGCACCATAGCCATCATAGC | TGTCCAGCATTCCACCAGTA |
| *Pdha1* | GAAATGTGACCTTCATCGGCT | TGATCCGCCTTTAGCTCCATC |
| *Hat1* | ACACCAACACAGCAATCGAG | TGTAACCGAAAGCAGTTTCATCA |
| *p300* | AGCCAAGCGGCCTAAACTC | CGCCACCATTGGTTAGTCCC |
| *Hdac1* | ACCACCAGAGGGTGCTCTAT | GCAGTGGGTAGTTCACAGCA |
| *Hdac2* | GAGTACAGTAAGCAGATGCAGAG | GATGTAGTCCTCCAGCCCAA |
| *Hdac3* | TGGCTACACTGTCCGAAATG | CTGGCGTGAGTTCTGATTCT |
| *Hdac4* | ACAGAAACTGGACAGCTCGC | CCACTACACAGCCTACAGCC |
| *Hdac5* | CCAGCAAGCATTCTACAACGAT | TGTCAGGTATTCCACATCTCCAA |
| **Gene** | **Forward primer sequence (5'→3')** | **Reverse primer sequence (5'→3')** |
| *Hdac6* | TGCTCTAGTTCTAGGCTTGTCAT | AGGTAGTGCTGCTATGGTCTT |
| *Hdac7* | CTGGCTGCTTTCAGGATAGTG | TTCATCAACTGCTGCGTCAT |
| *Hdac8* | ATCAACTGGTCTGGAGGGTG | CAAATTTCCGTCGCAATCGT |
| *Hdac9* | CCACTCAATGCTTCTAACTCACT | CAATGCTACTGCCATACTGACT |
| *Hdac10* | TAGCAACCACAACTGCCCTC | GGTCGATCCAGTTGTCCCAG |
| *Hdac11* | GCATTGTGAAGAGGGATGAAGT | GATGGAGTCGGCGATAATACG |
| *Sirt1* | ACCACCAAATCGTTACATATTCCA | CCGTATCATCTTCCAAGCCATT |
| *Sirt2* | GAACCCTTCTTTGCCCTTGC | AGCGTGTCTATGTTCTGCGT |
| *Sirt3* | AGCTACATGCACGGTCTGTC | AATGTCGGGTTTCACAACGC |
| *Sirt4* | GTACTCTGGTTACAGGTTCATCC | GGGTCTATTAAAGGCAGCAACT |
| *Sirt5* | AACCTGGATCCTGCCATTCTG | GGTCCGGGAAAATGAAACCT |
| *Sirt6* | GGACCTGATGCTCGCTGATG | GGTTGCAGGTTGACAATGACC |
| *Sirt7* | AGAACTGTGATGGGCTCCAC | CTGTCAGGTGTCGGTGAAGG |
| *Grin1* | ATGGGTTCGGTATCAAGAATGTG | GCATCCTTGTGTCGCTTGTA |
| *Grin2a* | ATTGGGAGCGGGTACATCTT | CCGTCACCAACAAACTGGAG |
| *Grin2b* | TGTCATGGTATCACGCAGCA | GCTCTCTGCCATCAGCTAGG |
| *Gria1* | TCCTCATACACAGCCAACCT | AGACCTCCTGAAGAACTCCTTAG |
| *Gria2* | TCTCCTCCTACACGGCTAAC | GCACTCCTCATATAAGTCCACATT |
| *Gria3* | GTCTTCGCTTACATTGGAGTCA | GGTCACGAGGTTCTTCATTGT |
| *β-actin* | TGTCCACCTTCCAGCAGATGT | AGCTCAGTAACAGTCCGCCTAG |
| *Grin1 promoter* | ACGATTGGTGGCTTGTATGAC | ATAGTGAGGGCAGGGAGAAG |
| *Grin2a promoter* | AAGGCAGATATGGTGGTATGAAC | TGTGTATCTTAGTCTGGCTGTCA |
| *Grin2b promoter* | CTTCACTGTGTCCTGGCTTAG | GGAATGTGGTCACTGCTCTC |
| *Gria1 promoter* | AGAGTGATGACCTTTAAGCCAGAT | CGTGAAGCAGGAGCAATGTG |
| *Gria2 promoter* | GCAACCAACCACTGAAACAATC | TCTGCTGTTCACTCCTGGAA |
| *Gria3 promoter* | AATTGTCACTGCCTGCCATT | CTCTCACCATCCTTCCTCACA |
|  |  |  |
