# Supplementary figures and images for: ACSS2-dependent histone acetylation improves cognition in mouse model of Alzheimer’s disease
Source: Mol Neurodegener. 2023 Jul 12;18:47. doi: 10.1186/s13024-023-00625-4 (PMC10339567; doi:10.1186/s13024-023-00625-4)

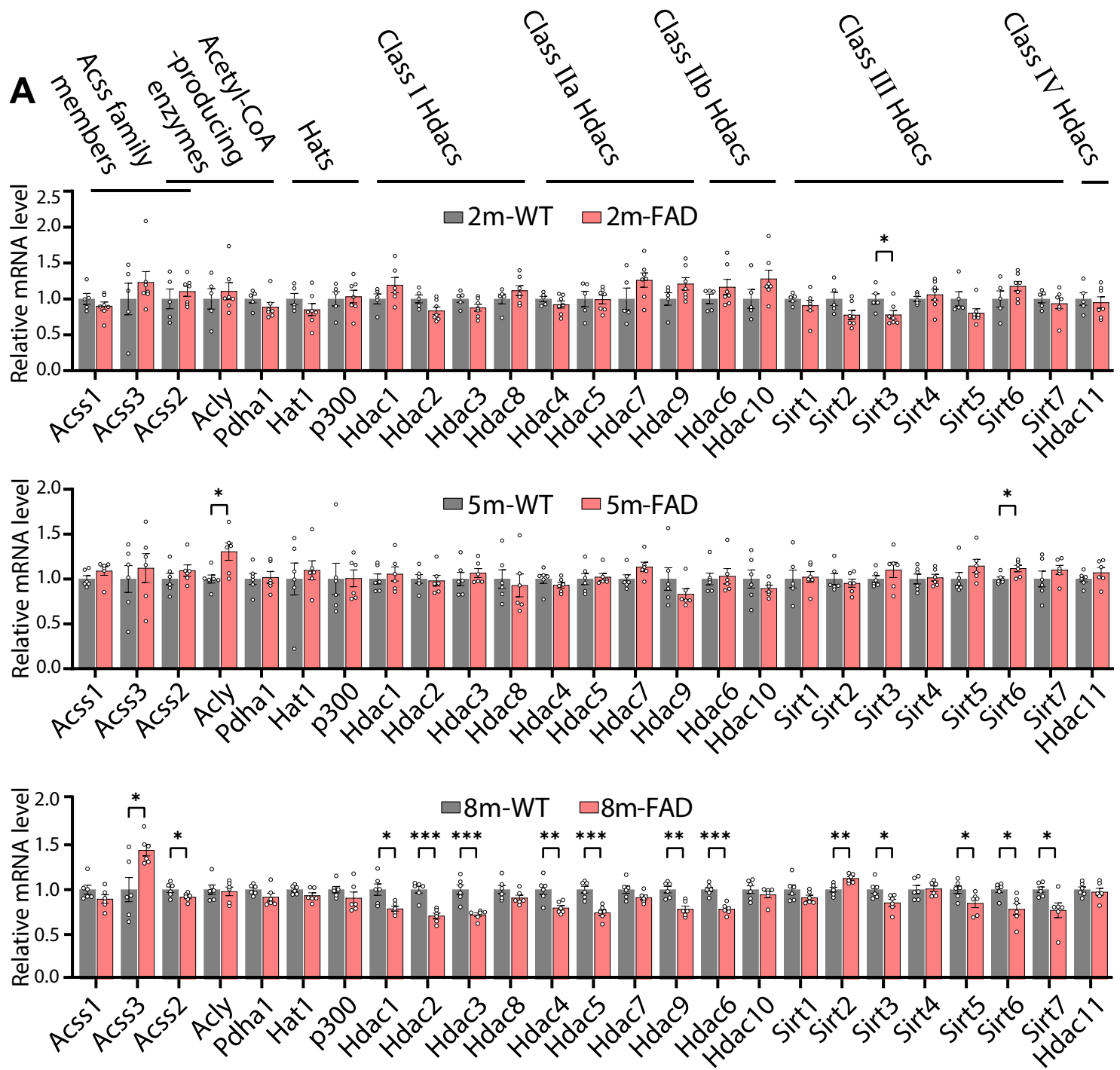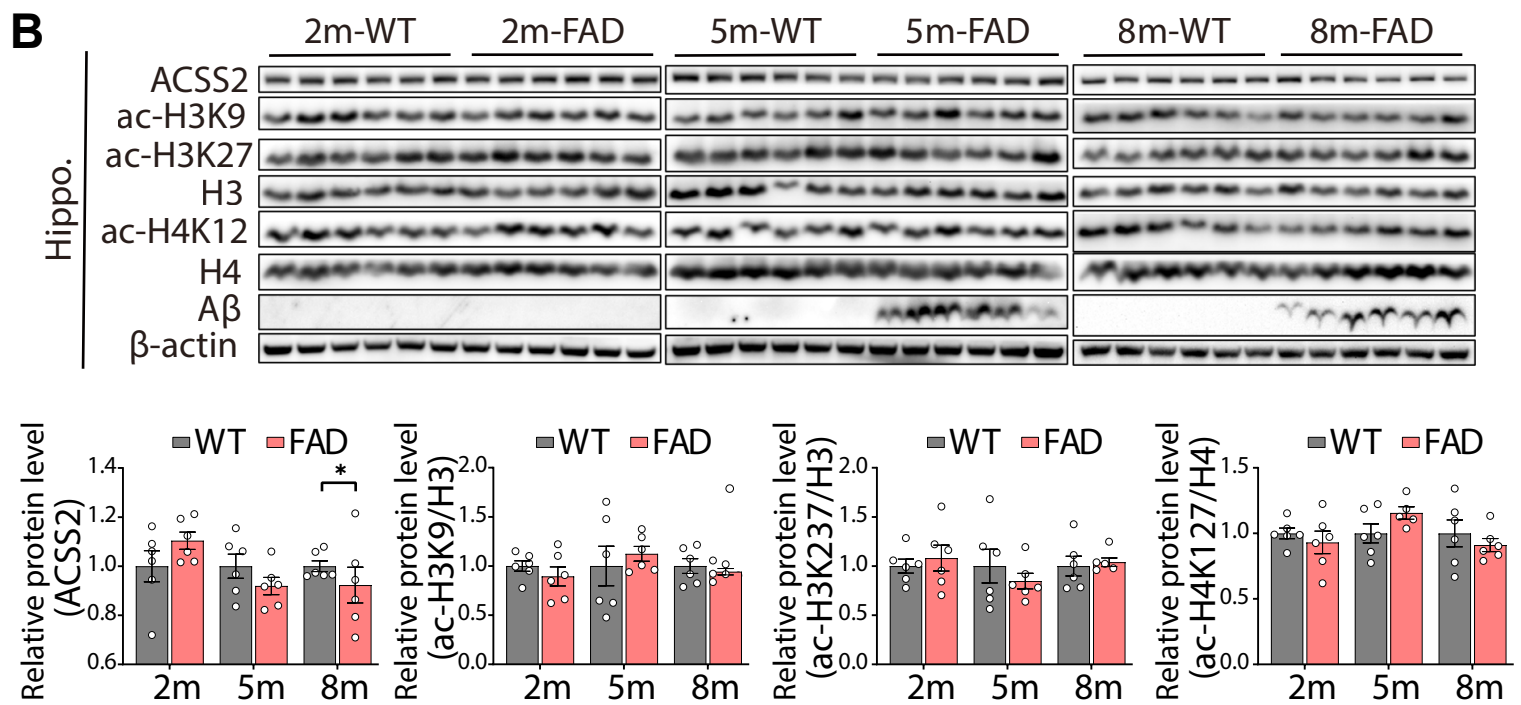

Supplement: Supplementary file 2 — Additional file 2: Fig. S1 The levels of enzymes involved in regulating histone acetylation and acetyl-CoA level in the hippocampi of 2-, 5- and 8-month-old 5×FAD mice. A The RT-qPCR analysis of Acss1, Acss3, Acss2, Acly, Pdha1, Hats, and Hdacsin the hippocampus of 2-, 5- and 8-month-old WT and 5×FAD mice. B Immunoblots and western blot analyses of ACSS2, ac-H3K9/H3, ac-H3K27/H3, ac-H4K12/H4, and Aβin the hippocampi of 2-, 5- and 8-month-old WT and 5×FAD mice. Data are expressed as mean ± SEM. * P < 0.05 by the unpaired two-tailed t-test. [file 13024_2023_625_MOESM2_ESM.pdf]

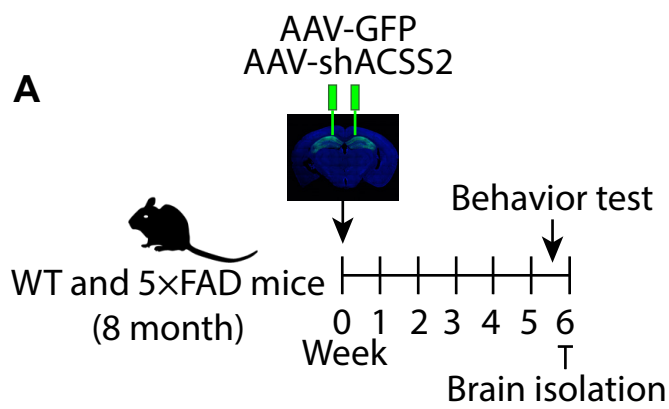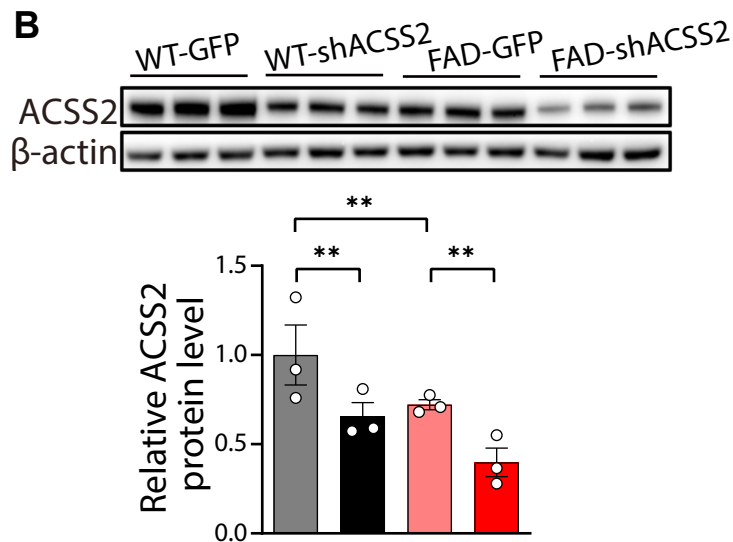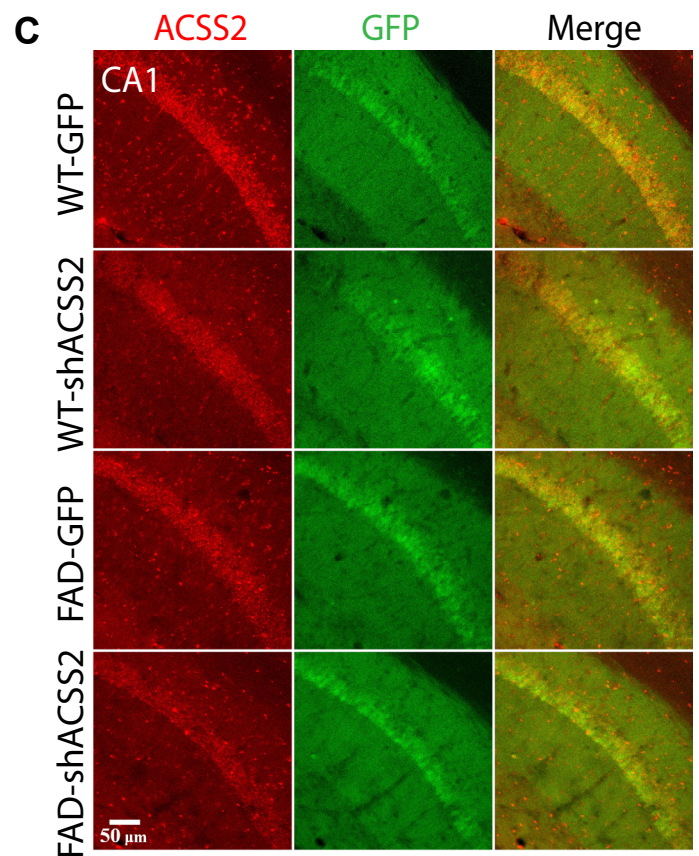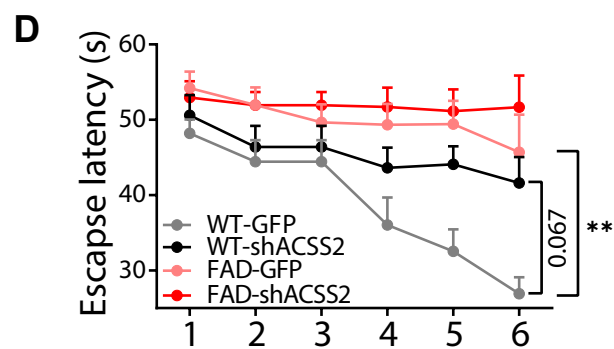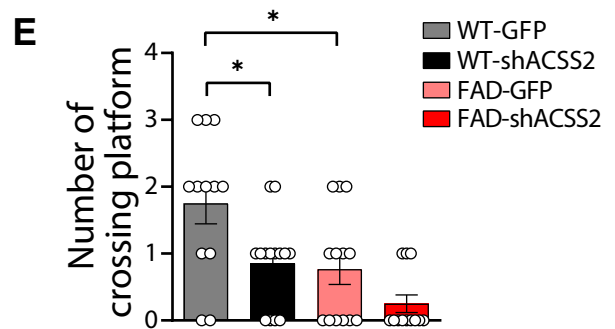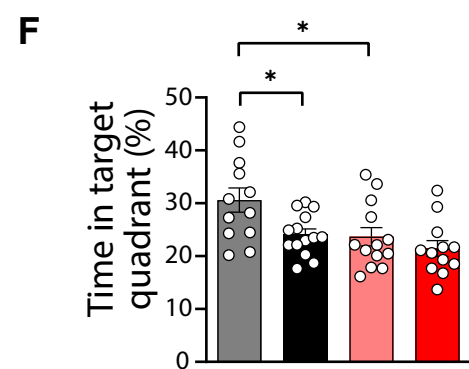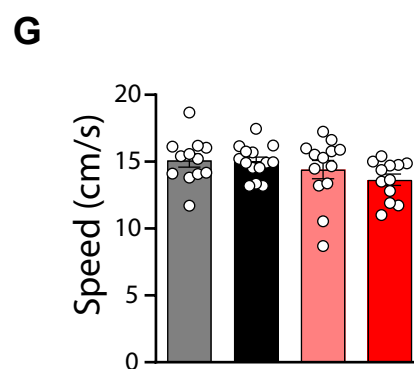

Supplement: Supplementary file 3 — Additional file 3: Fig. S2. The impaired spatial learning and memory in WT and 5×FAD mice by ACSS2 knockdown. A The time schedule of the experimental procedure of ACSS2 knockdown. B, C Knockdown efficiency of shAcss2 was examined by western blot and immunofluorescence. Immunoblots and western blot analysis of ACSS2 in the dorsal hippocampus of mice injected with either GFP control virus or ACSS2 knockdown virus. Representative images of ACSS2with GFP in the dorsal hippocampus from mice injected with either GFP control virus or ACSS2 knockdown virus. D-GACSS2 knockdown mice were tested in the Morris water maze. Escape latency to the platform position during the training trailsand the probe trial. The number of platform-position crossings, the percentage of time spent in the target quadrant, and the speedin the probetrial. n= 12, 14, 13, and 12 mice for WT-GFP, WT-shAcss2, FAD-GFP, and FAD-shAcss2, respectively. Data are expressed as mean ± SEM. Statistical significance was calculated by two-way ANOVAand three-way ANOVAfollowed by the Tukey’s post-test, and by Scheirer-Ray-Hare test followed by the Dunn’s post-hot test. * P < 0.05, ** P< 0.01. [file 13024_2023_625_MOESM3_ESM.pdf]

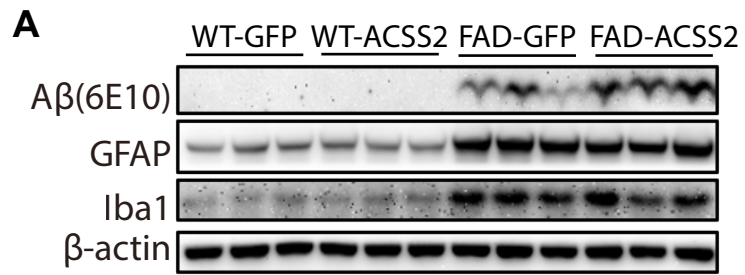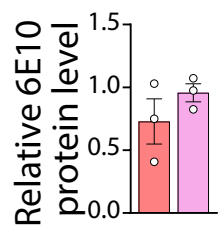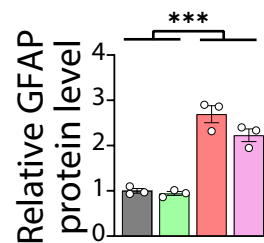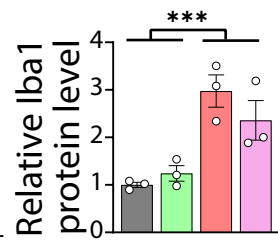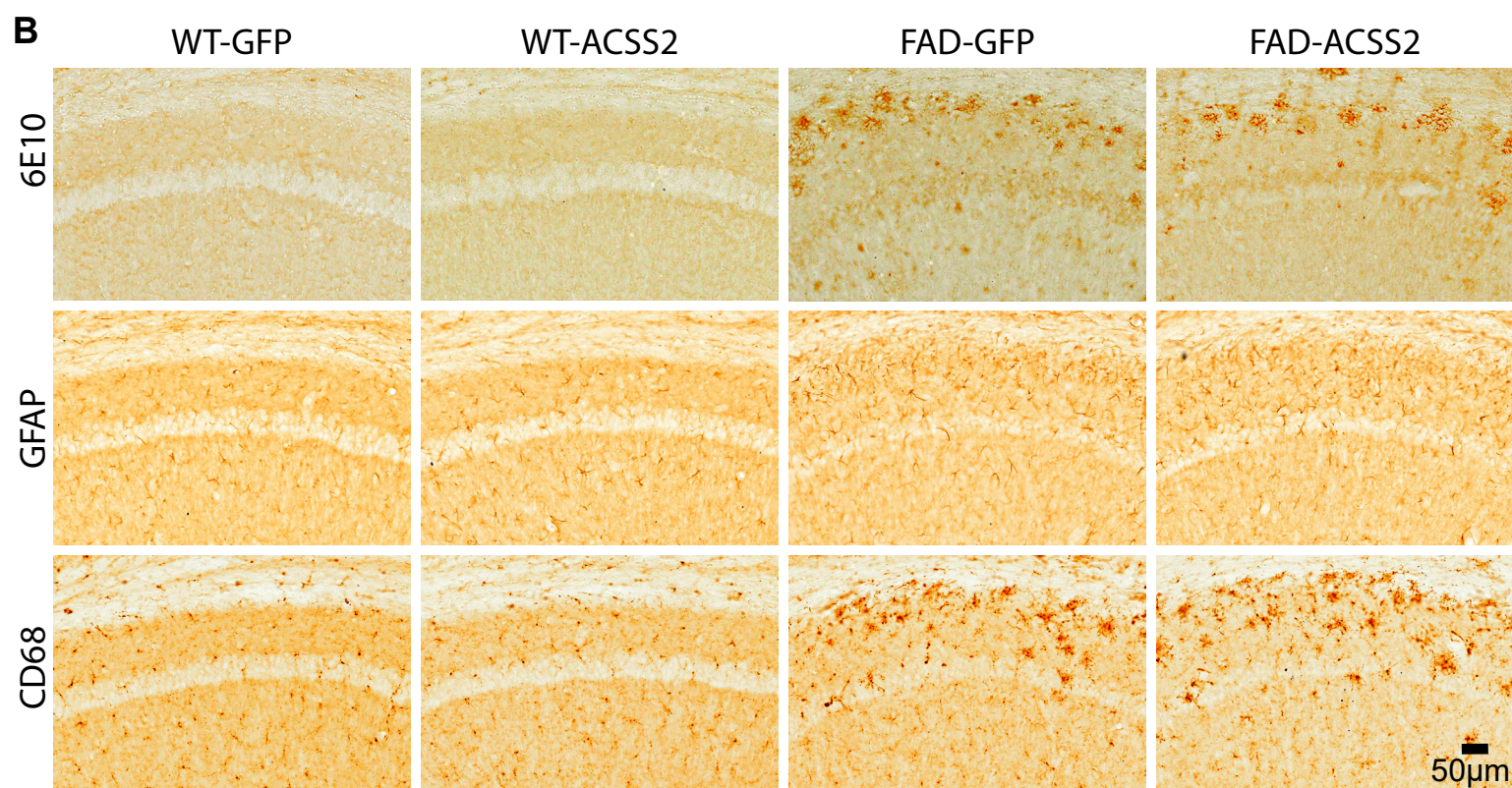

Supplement: Supplementary file 4 — Additional file 4: Fig. S3. The minimal effect of the ACSS2 upregulation on Aβ pathology and gliosis in middle-aged 5×FAD mice.A Representative immunoblots and quantitative analyses of Aβ, GFAP, and Iba1in the dorsal hippocampi of the WT-GFP, WT-ACSS2, FAD-GFP, and FAD-ACSS2 mice. n = 3 per group. B Representative images of immunohistochemical staining for Aβ , GFAP, and CD68in the dorsal hippocampus. Data are expressed as mean ± SEM. Statistical significance was calculated by the unpaired two-tailed t-testand two-way ANOVA followed by the Tukey’s post-test. ** P < 0.01, *** P < 0.001. [file 13024_2023_625_MOESM4_ESM.pdf]
