# Supplementary material for: ACSS2-dependent histone acetylation improves cognition in mouse model of Alzheimer’s disease
Source: Mol Neurodegener. 2023 Jul 12;18:47. doi: 10.1186/s13024-023-00625-4 (PMC10339567; doi:10.1186/s13024-023-00625-4)
Supplement: Supplementary file 5 — Additional file 5. [file 13024_2023_625_MOESM5_ESM.pdf]

Fig. 1A

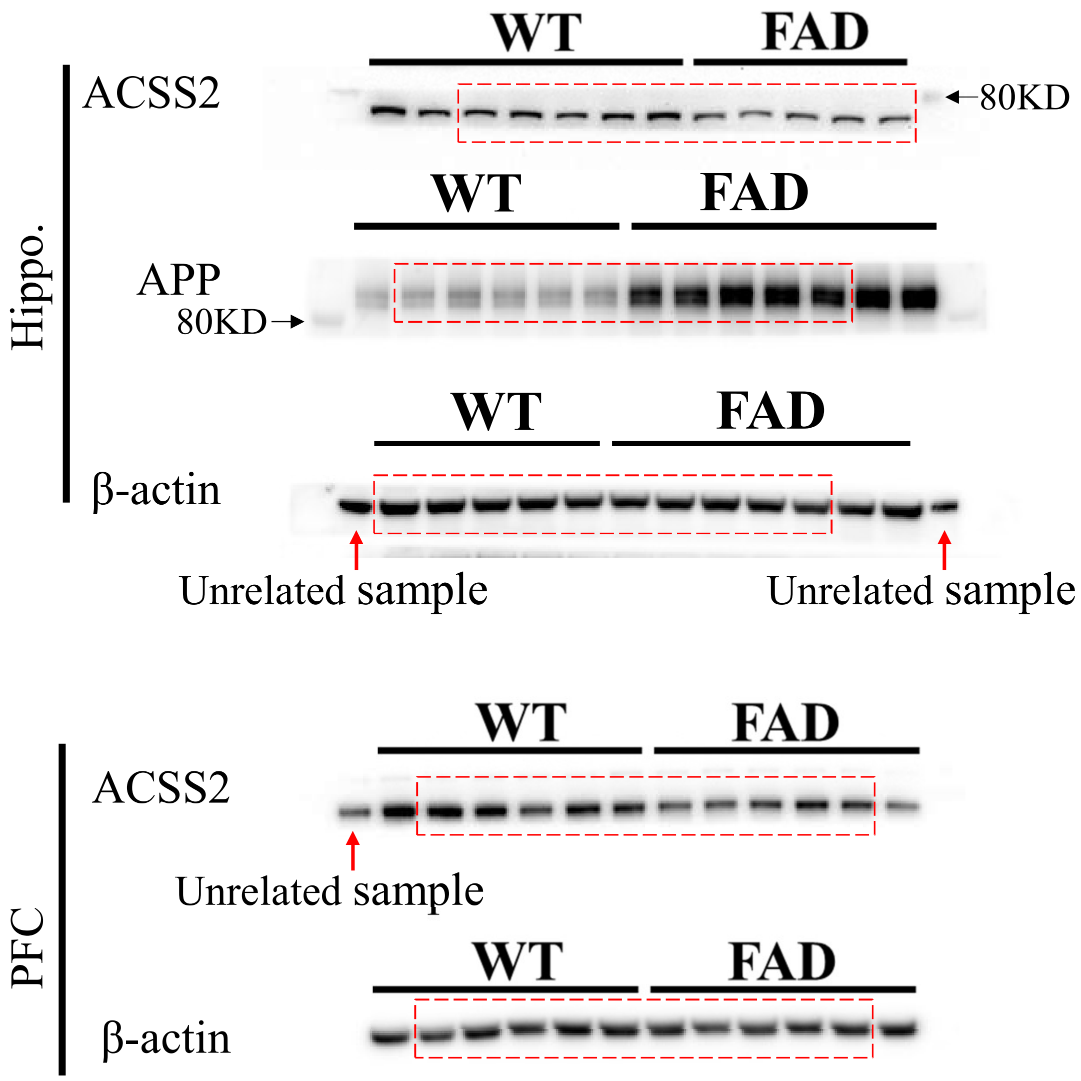

WT
FAD
ACSS2
80KD
Hippo.
WT
FAD
APP
80KD
WT
FAD
β-actin
Unrelated sample
Unrelated sample
WT
FAD
ACSS2
Unrelated sample
PFC
WT
FAD
β-actin

Fig. 1C

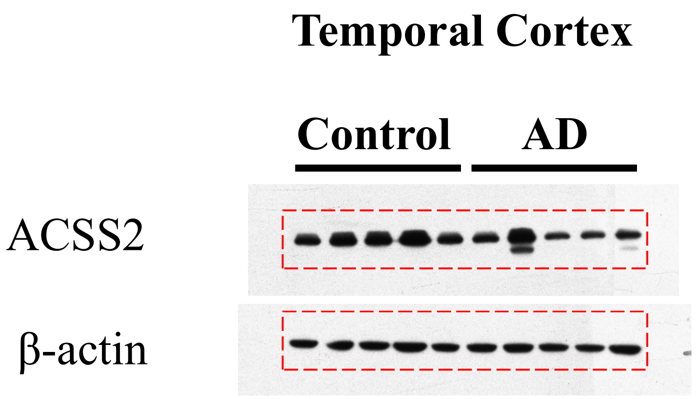

Fig.1D

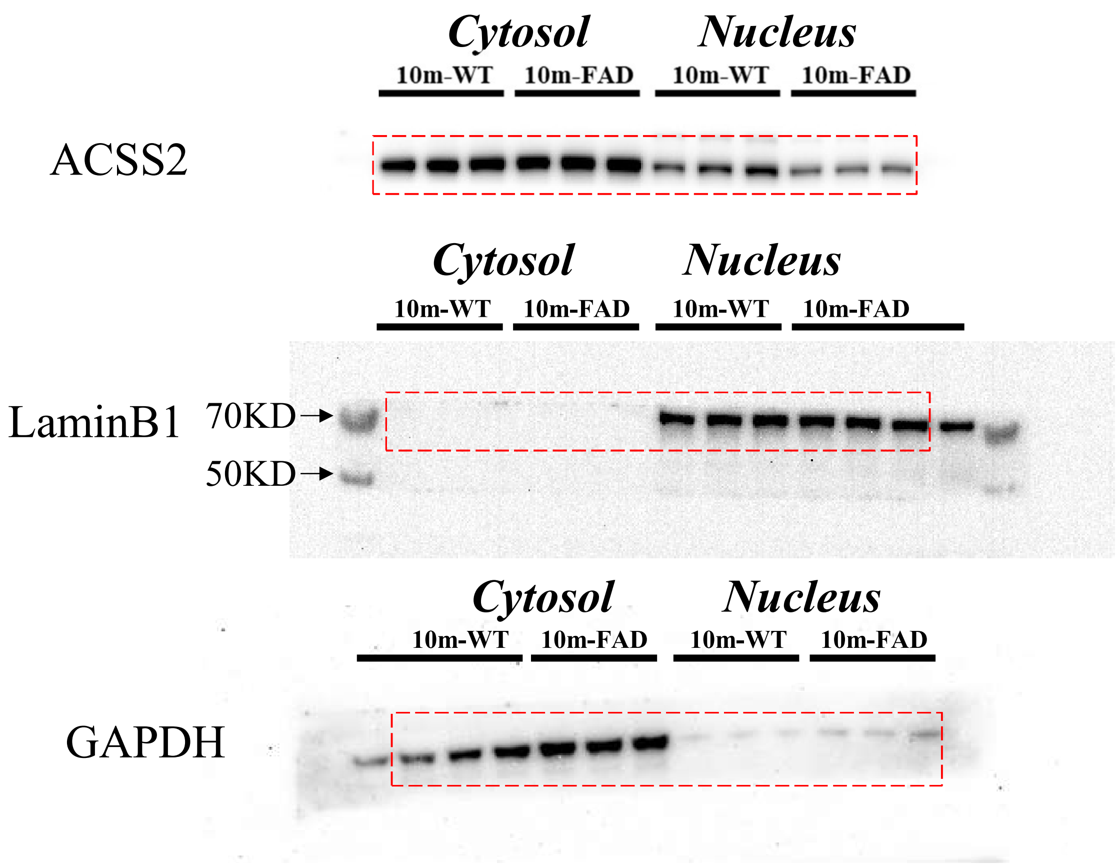
Cytosol
Nucleus
10m-WT
10m-FAD
10m-WT
10m-FAD
ACSS2
Cytosol
Nucleus
10m-WT
10m-FAD
10m-WT
10m-FAD
LaminB1
70KD
50KD
Cytosol
Nucleus
10m-WT
10m-FAD
10m-WT
10m-FAD
GAPDH

Fig. 1F

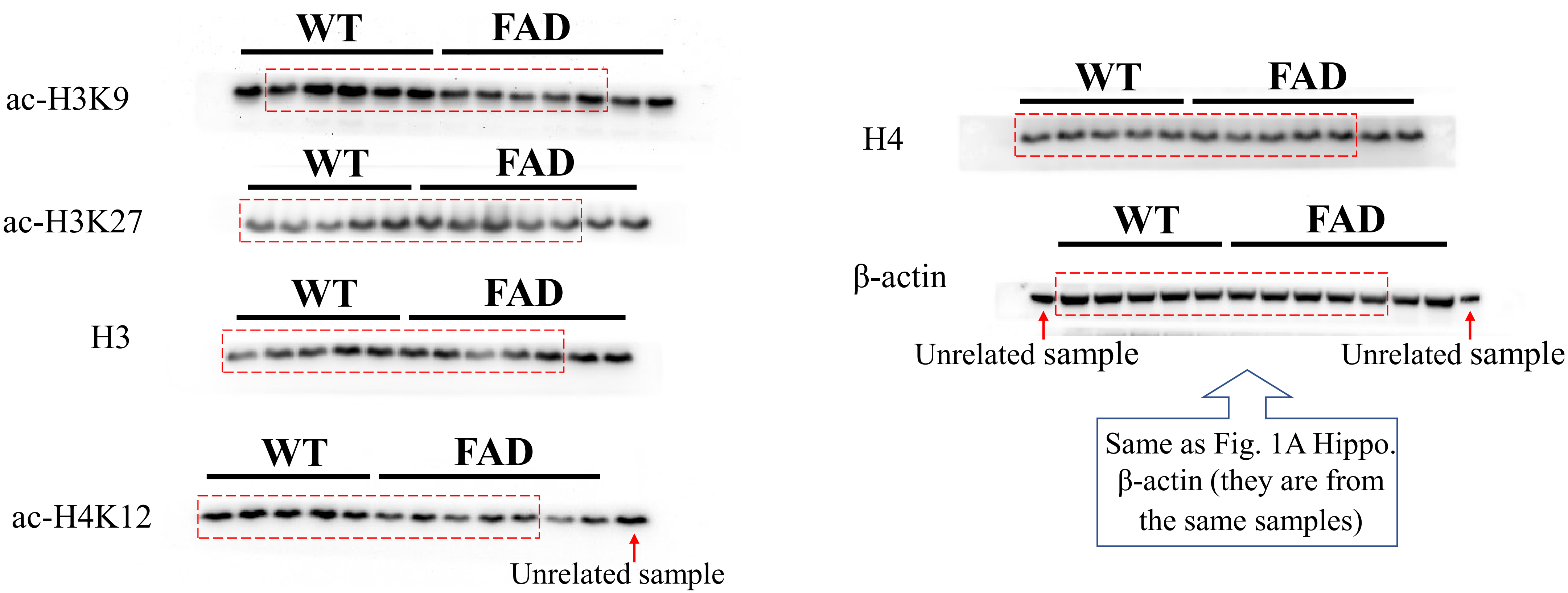
WT
FAD
ac-H3K9
ac-H3K27
H3
ac-H4K12
Unrelated sample
H4
β-actin
Unrelated sample
Unrelated sample
Same as Fig. 1A Hippo. β-actin (they are from the same samples)

Fig. 2A

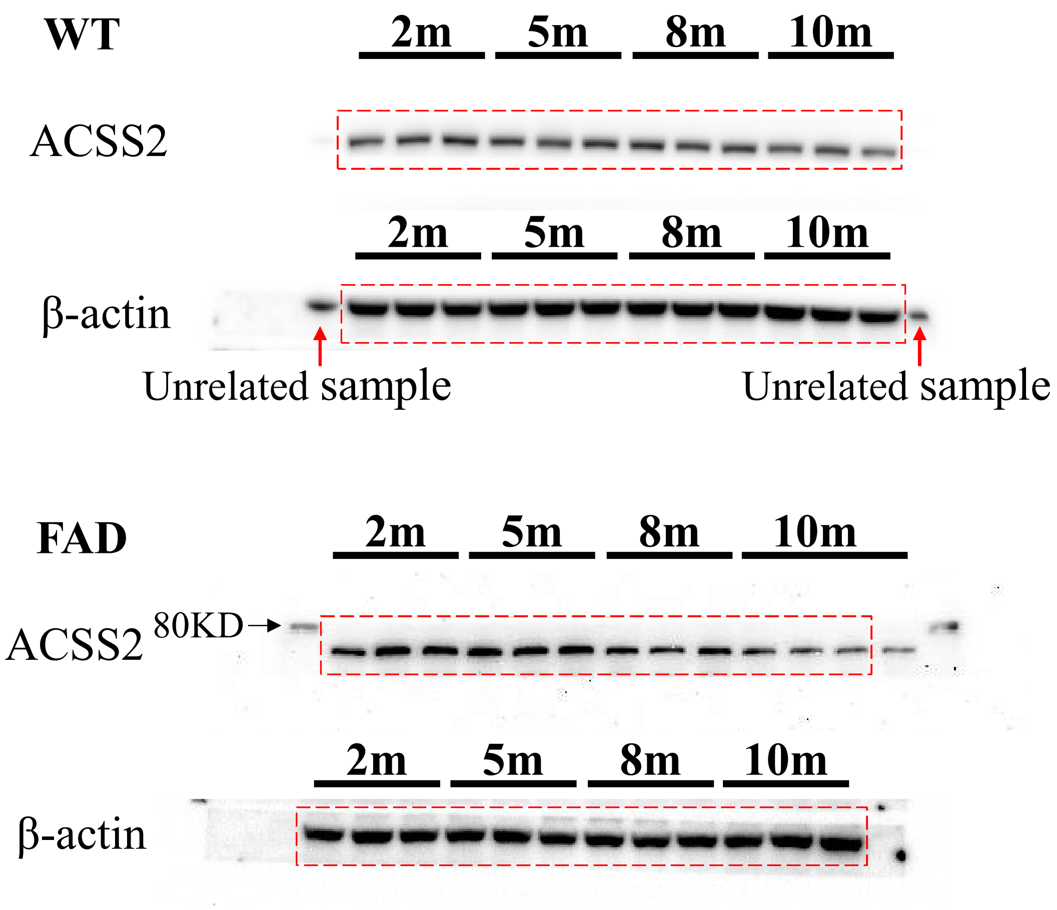
WT
2m
5m
8m
10m
ACSS2
2m
5m
8m
10m
β-actin
Unrelated sample
Unrelated sample
FAD
2m
5m
8m
10m
ACSS2
80KD
2m
5m
8m
10m
β-actin

Fig. 3B

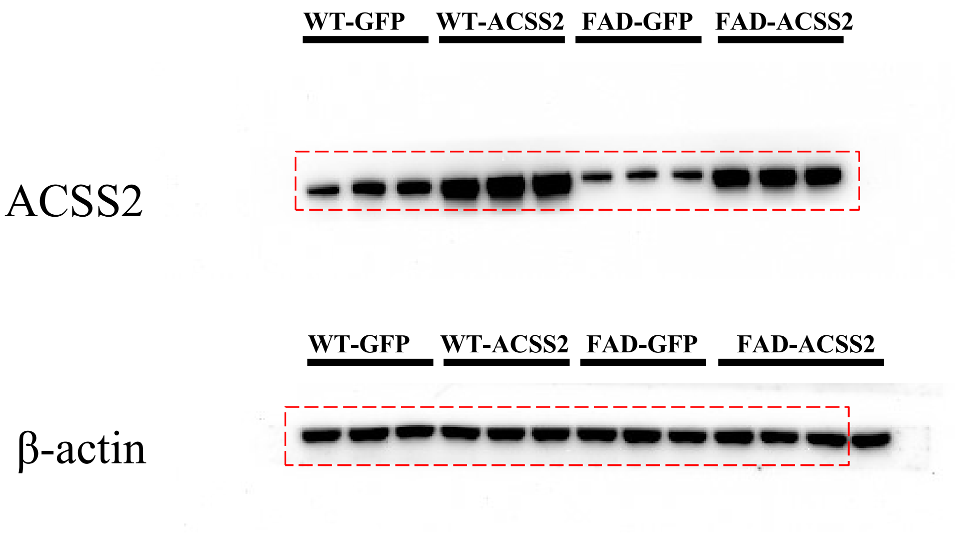
WT-GFP
WT-ACSS2
FAD-GFP
FAD-ACSS2
ACSS2
WT-GFP
WT-ACSS2
FAD-GFP
FAD-ACSS2
β-actin

Fig. 5B

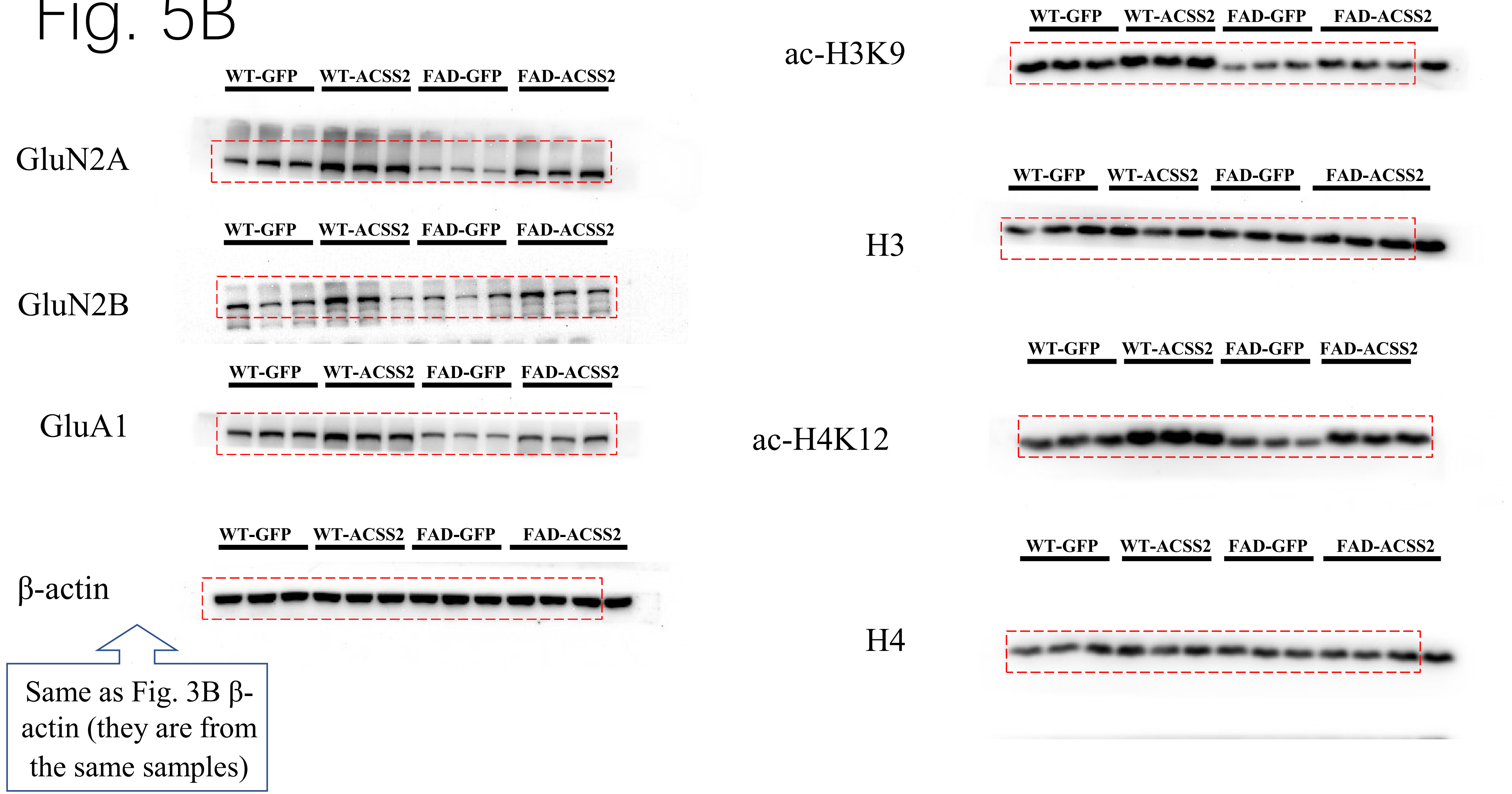
WT-GFP
WT-ACSS2
FAD-GFP
FAD-ACSS2
GluN2A
GluN2B
GluA1
β-actin
Same as Fig. 3B β-actin (they are from the same samples)
ac-H3K9
H3
ac-H4K12
H4

Fig. 6A

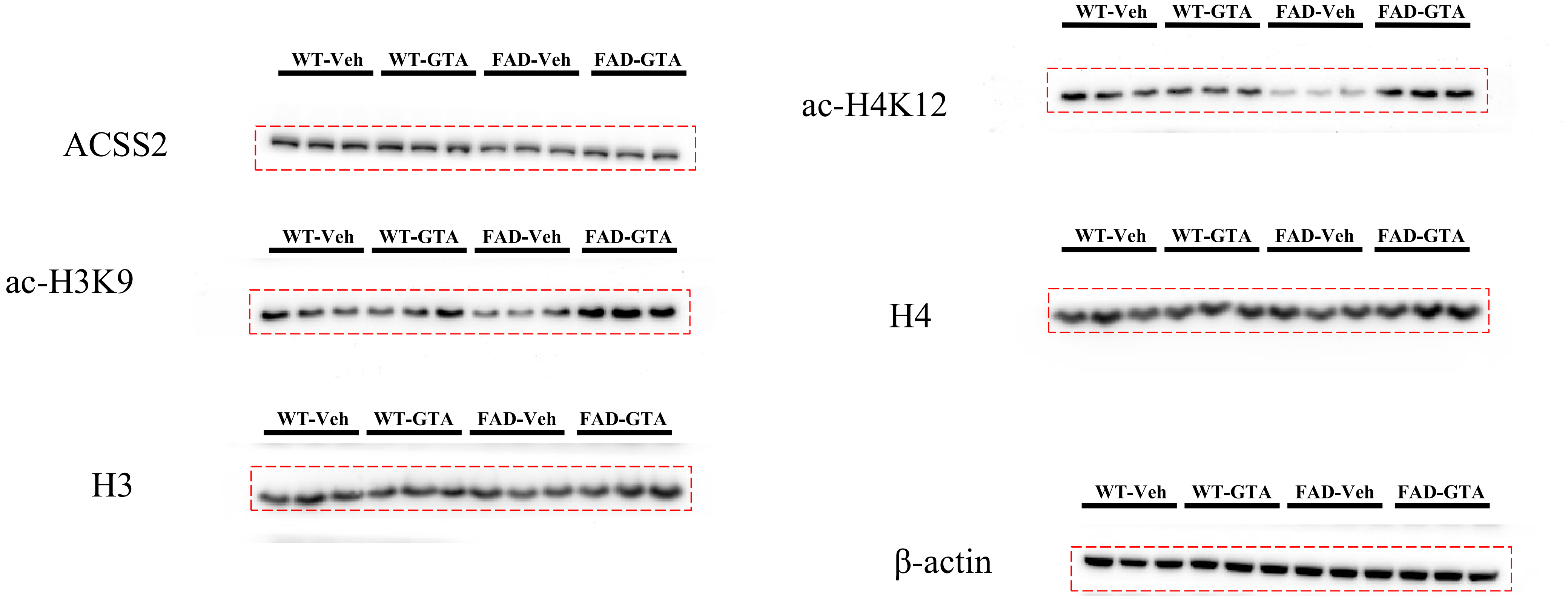

WT-Veh
WT-GTA
FAD-Veh
FAD-GTA
ACSS2
ac-H3K9
H3
ac-H4K12
H4
β-actin

Fig. 6D

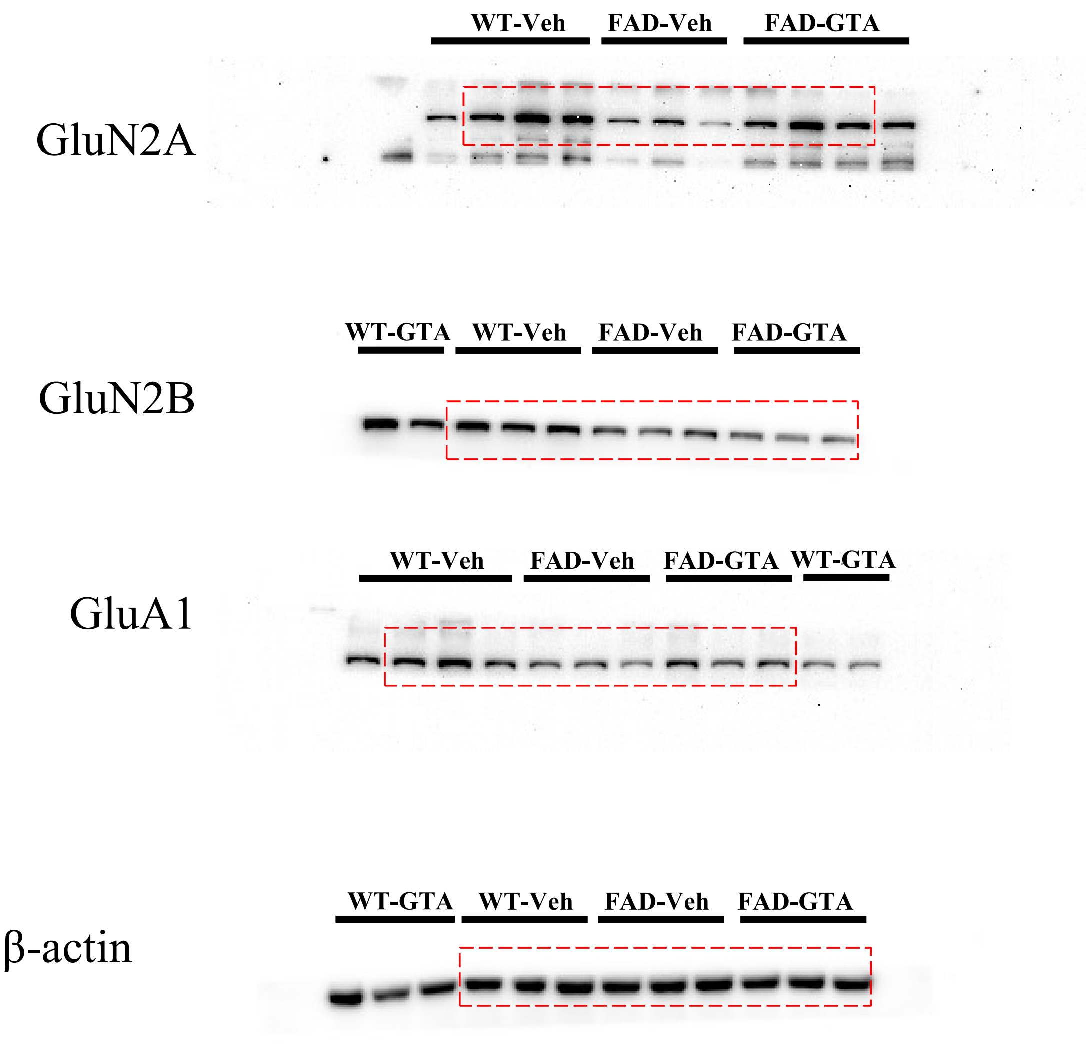
WT-Veh
FAD-Veh
FAD-GTA
GluN2A
WT-GTA
WT-Veh
FAD-Veh
FAD-GTA
GluN2B
WT-Veh
FAD-Veh
FAD-GTA
WT-GTA
GluA1
WT-GTA
WT-Veh
FAD-Veh
FAD-GTA
β-actin

Fig. 7G and Fig. 7I

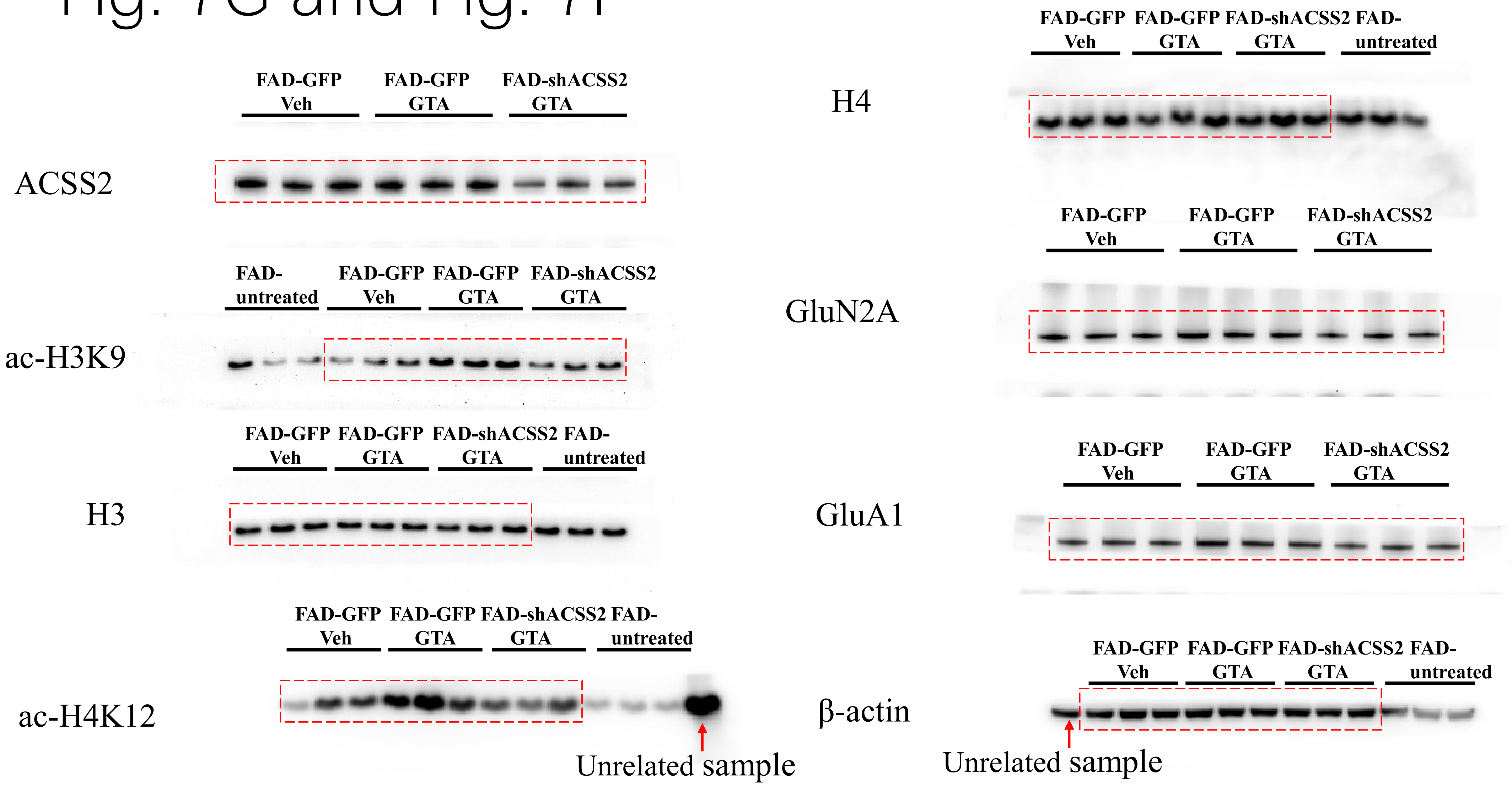

Fig. S1B-2m

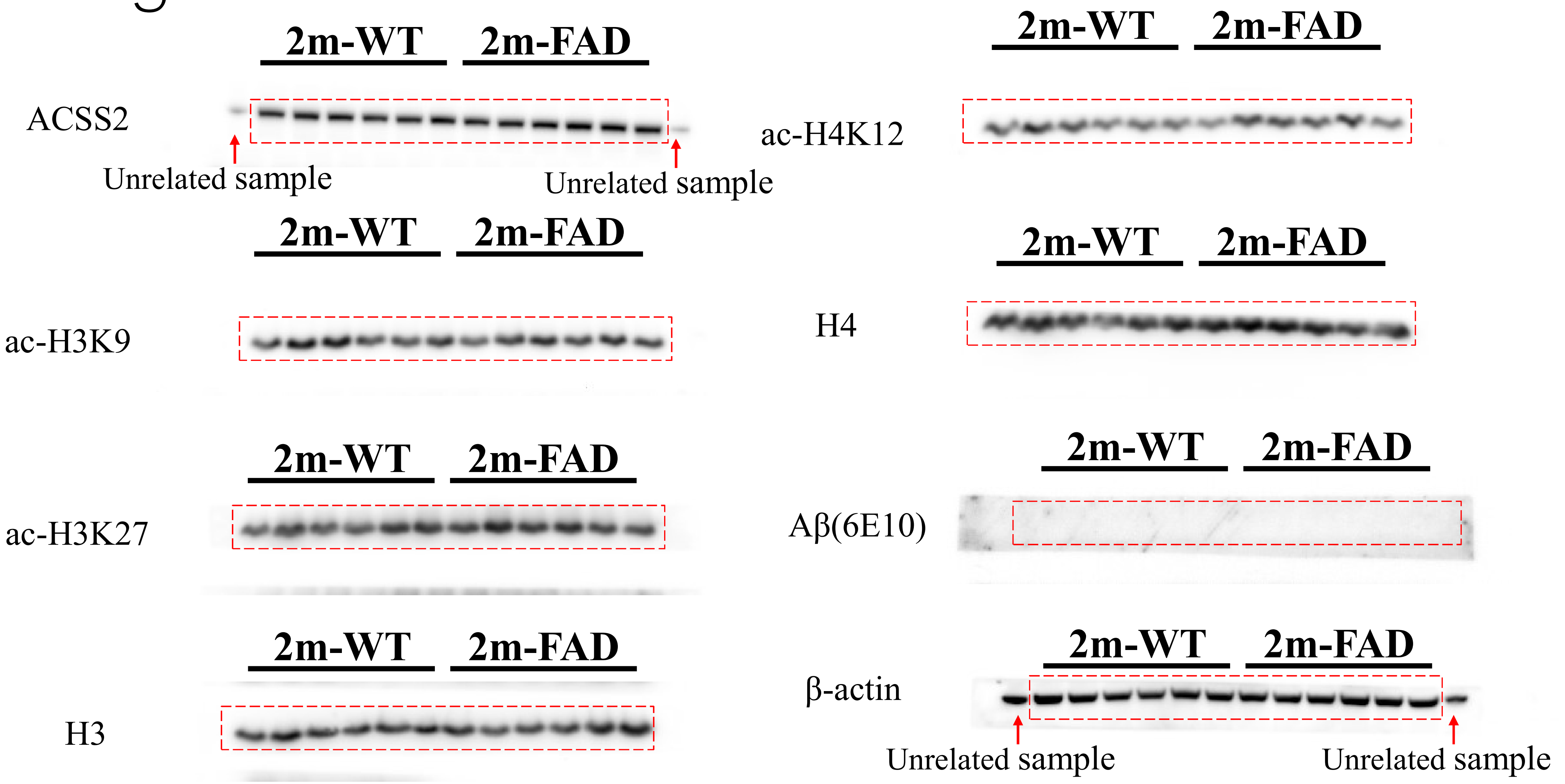
2m-WT
2m-FAD
ACSS2
Unrelated sample
Unrelated sample
ac-H4K12
ac-H3K9
H4
ac-H3K27
Aβ(6E10)
H3
β-actin
Unrelated sample
Unrelated sample

Fig. S1B-5m

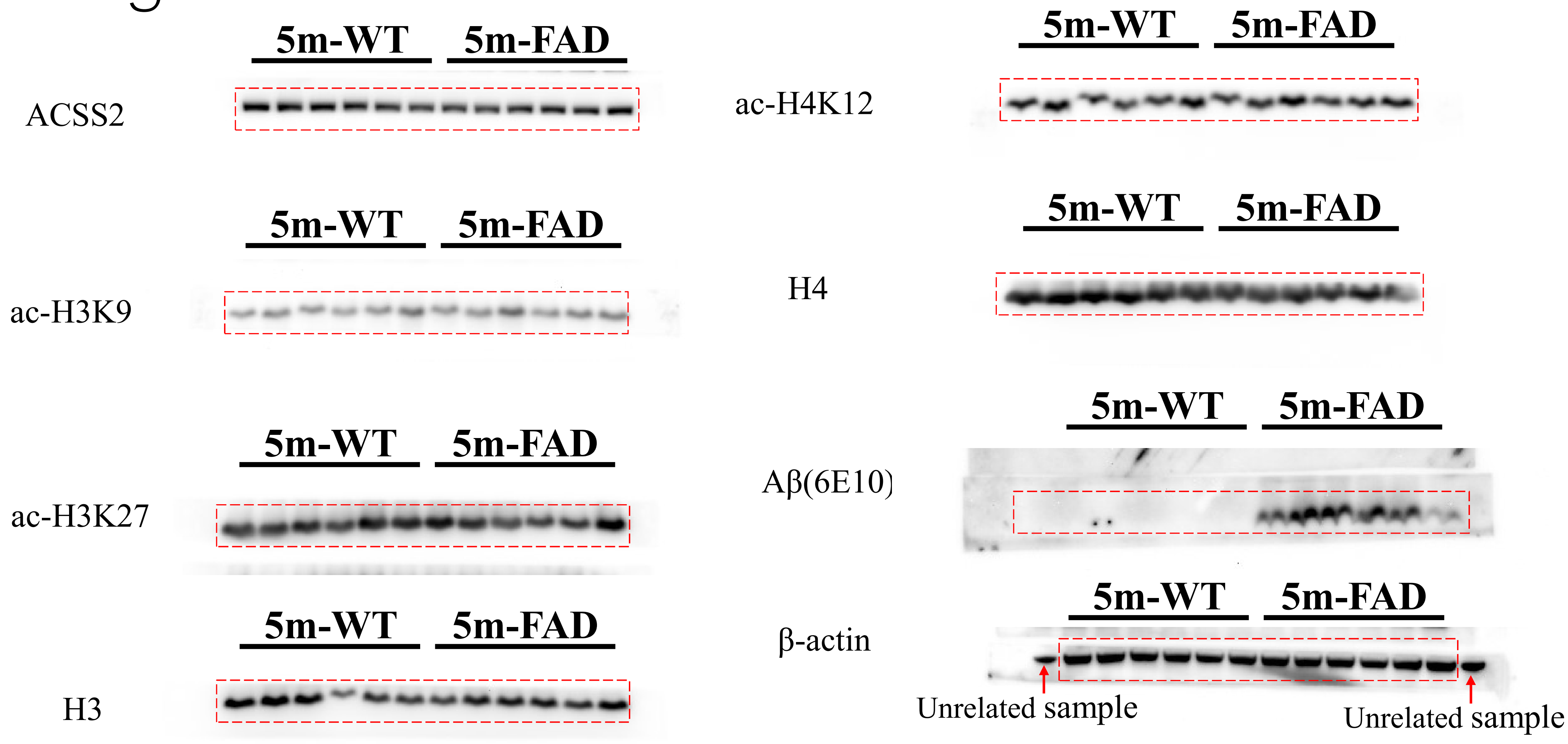
5m-WT
5m-FAD
ACSS2
ac-H3K9
ac-H3K27
H3
ac-H4K12
H4
Aβ(6E10)
β-actin
Unrelated sample
Unrelated sample

Fig. S1B-8m

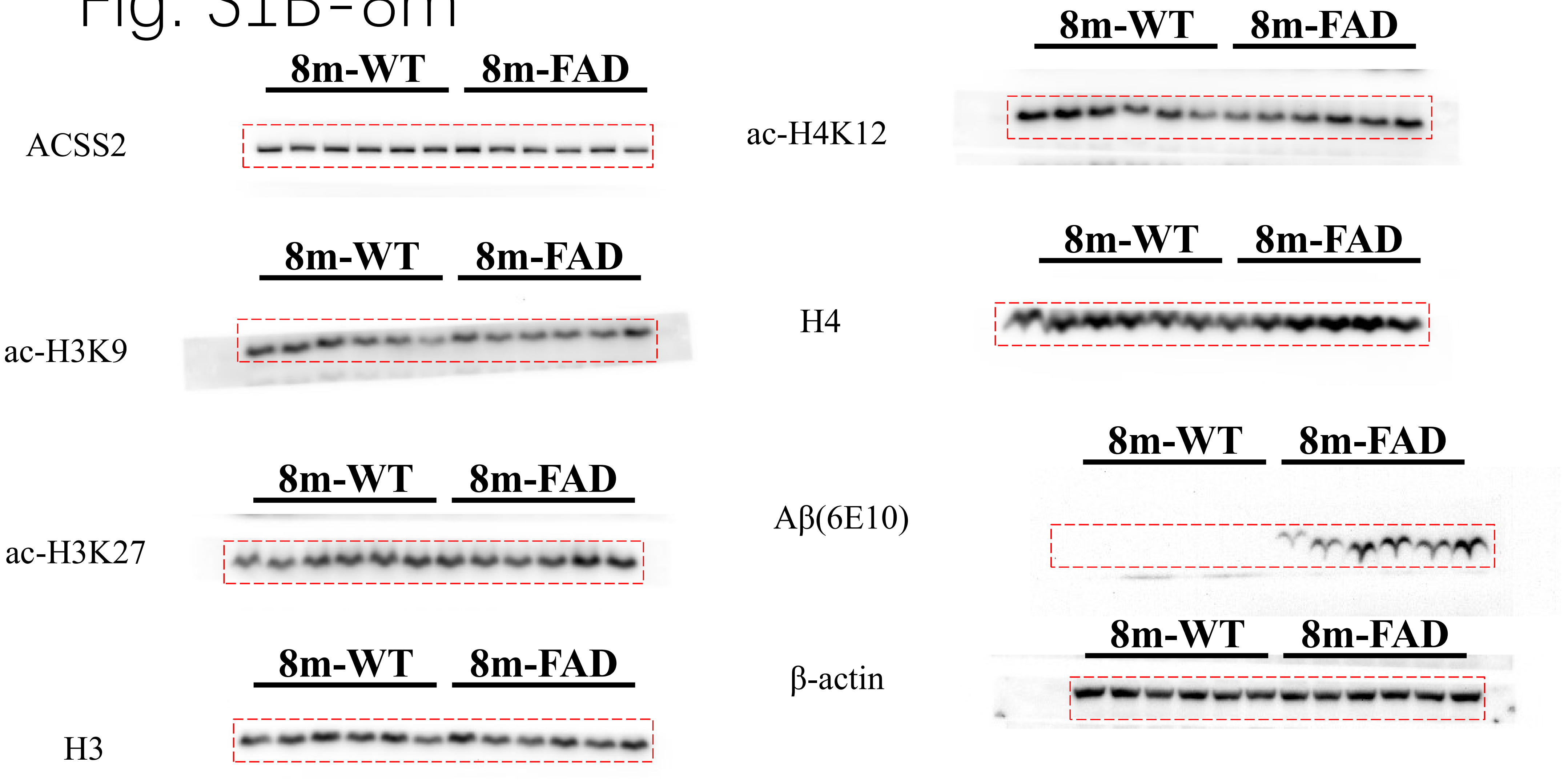
8m-WT
8m-FAD
ACSS2
8m-WT
8m-FAD
ac-H3K9
8m-WT
8m-FAD
ac-H3K27
8m-WT
8m-FAD
H3
8m-WT
8m-FAD
ac-H4K12
8m-WT
8m-FAD
H4
8m-WT
8m-FAD
Aβ(6E10)
8m-WT
8m-FAD
β-actin

Fig. S2B

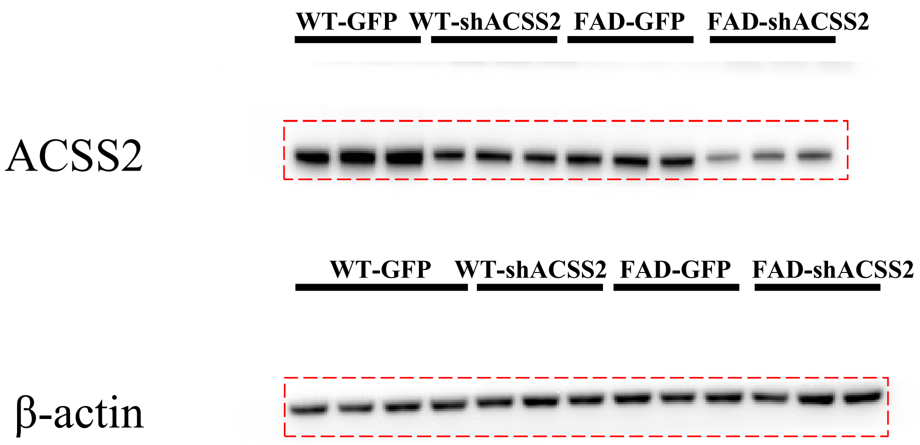
WT-GFP
WT-shACSS2
FAD-GFP
FAD-shACSS2
ACSS2
WT-GFP
WT-shACSS2
FAD-GFP
FAD-shACSS2
β-actin

Fig. S3A

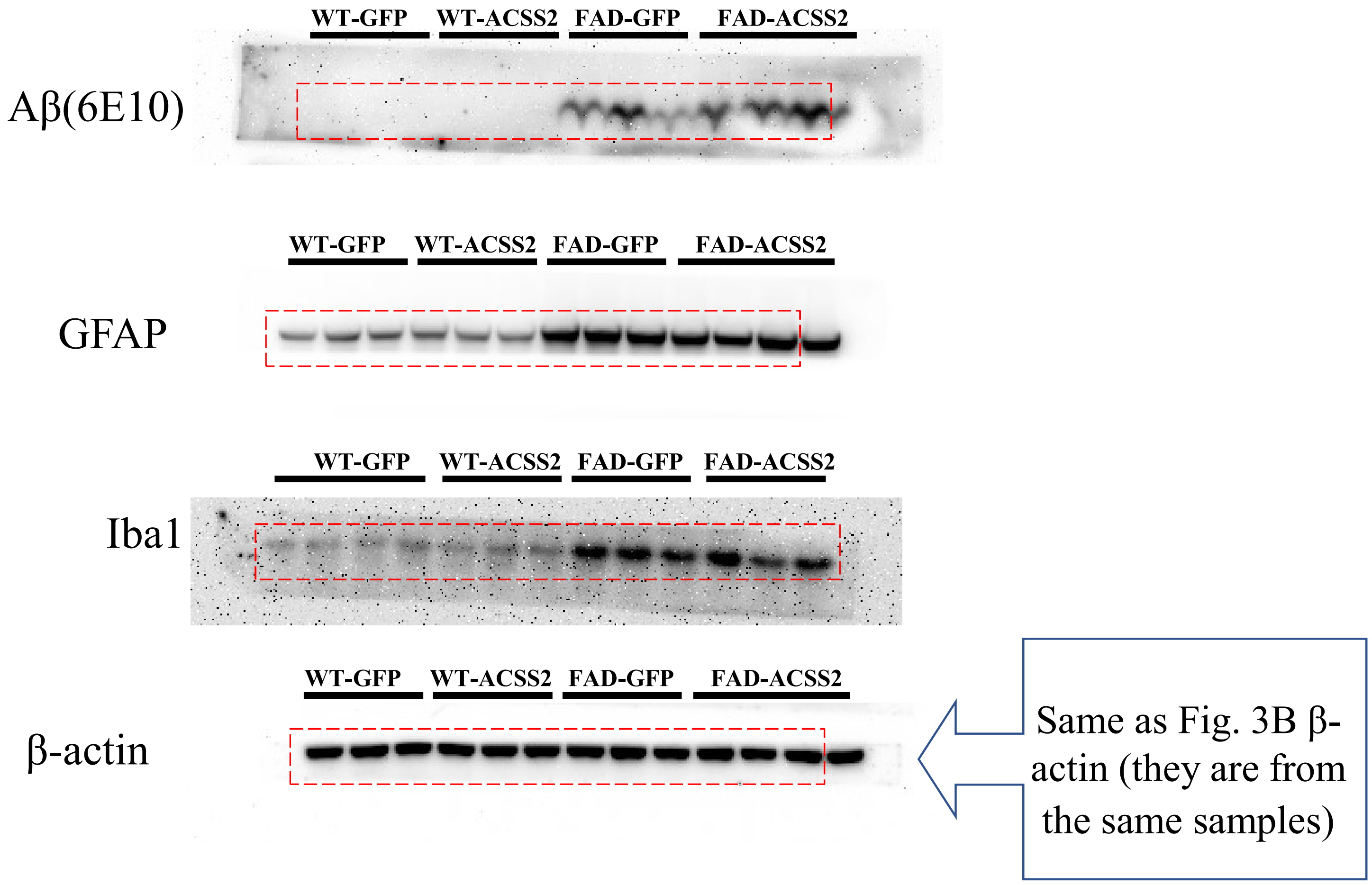
WT-GFP
WT-ACSS2
FAD-GFP
FAD-ACSS2
Aβ(6E10)
WT-GFP
WT-ACSS2
FAD-GFP
FAD-ACSS2
GFAP
WT-GFP
WT-ACSS2
FAD-GFP
FAD-ACSS2
Iba1
WT-GFP
WT-ACSS2
FAD-GFP
FAD-ACSS2
β-actin
Same as Fig. 3B β-actin (they are from the same samples)
